# A Splice Region Variant in *LDLR* Lowers Non-high Density Lipoprotein Cholesterol and Protects against Coronary Artery Disease

**DOI:** 10.1371/journal.pgen.1005379

**Published:** 2015-09-01

**Authors:** Solveig Gretarsdottir, Hannes Helgason, Anna Helgadottir, Asgeir Sigurdsson, Gudmar Thorleifsson, Audur Magnusdottir, Asmundur Oddsson, Valgerdur Steinthorsdottir, Thorunn Rafnar, Jacqueline de Graaf, Maryam S. Daneshpour, Mehdi Hedayati, Fereidoun Azizi, Niels Grarup, Torben Jørgensen, Henrik Vestergaard, Torben Hansen, Gudmundur Eyjolfsson, Olof Sigurdardottir, Isleifur Olafsson, Lambertus A. Kiemeney, Oluf Pedersen, Patrick Sulem, Gudmundur Thorgeirsson, Daniel F. Gudbjartsson, Hilma Holm, Unnur Thorsteinsdottir, Kari Stefansson

**Affiliations:** 1 deCODE Genetics/Amgen, Inc., Reykjavik, Iceland; 2 School of Engineering and Natural Sciences, University of Iceland, Reykjavik, Iceland; 3 Faculty of Medicine, University of Iceland, Reykjavik, Iceland; 4 Radboud University Medical Center, Radboud Institute for Health Sciences, Nijmegen, The Netherlands; 5 Cellular and Molecular Endocrine Research Center, Research Institute for Endocrine Sciences, Shahid Beheshti University of Medical Sciences, Tehran, I. R. Iran; 6 Endocrine Research Center, Research Institute for Endocrine Sciences, Shahid Beheshti University of Medical Sciences, Tehran, I. R. Iran; 7 The Novo Nordisk Foundation Center for Basic Metabolic Research, Section of Metabolic Genetics, Faculty of Health and Medical Sciences, University of Copenhagen, Copenhagen, Denmark; 8 Research Centre for Prevention and Health, The Capital Region of Denmark, Copenhagen, Denmark; 9 Faculty of Medicine, University of Aalborg, Aalborg, Denmark; 10 Institute of Public Health and Medical Science, University of Copenhagen, Copenhagen, Denmark; 11 Faculty of Health Sciences, University of Southern Denmark, Odense, Denmark; 12 The Laboratory in Mjodd, RAM, Reykjavik, Iceland; 13 Department of Clinical Biochemistry, Akureyri Hospital, Akureyri, Iceland; 14 Department of Clinical Biochemistry, Landspitali, National University Hospital, Reykjavik, Iceland; 15 Division of Cardiology, Department of Internal Medicine, Landspitali, National University Hospital of Iceland, Reykjavik, Iceland; Mount Sinai School of Medicine, UNITED STATES

## Abstract

Through high coverage whole-genome sequencing and imputation of the identified variants into a large fraction of the Icelandic population, we found four independent signals in the low density lipoprotein receptor gene (*LDLR*) that associate with levels of non-high density lipoprotein cholesterol (non-HDL-C) and coronary artery disease (CAD). Two signals are novel with respect to association with non-HDL-C and are represented by non-coding low frequency variants (between 2–4% frequency), the splice region variant rs72658867-A in intron 14 and rs17248748-T in intron one. These two novel associations were replicated in three additional populations. Both variants lower non-HDL-C levels (rs72658867-A, non-HDL-C effect = -0.44 mmol/l, *P*_*adj*_ = 1.1 × 10^−80^ and rs17248748-T, non-HDL-C effect = -0.13 mmol/l, *P*_*adj*_ = 1.3 × 10^−12^) and confer protection against CAD (rs72658867-A, OR = 0.76 and *P*_*adj*_ = 2.7 × 10^−8^ and rs17248748-T, OR = 0.92 and *P*_*adj*_ = 0.022). The *LDLR* splice region variant, rs72658867-A, located at position +5 in intron 14 (NM_000527:c.2140+5G>A), causes retention of intron 14 during transcription and is expected to produce a truncated LDL receptor lacking domains essential for function of the receptor. About half of the transcripts generated from chromosomes carrying rs72658867-A are characterized by this retention of the intron. The same variant also increases *LDLR* mRNA expression, however, the wild type transcripts do not exceed levels in non-carriers. This demonstrates that sequence variants that disrupt the LDL receptor can lower non-HDL-C and protect against CAD.

## Author Summary

Cholesterol levels in the bloodstream, in particular elevated low-density lipoprotein cholesterol (LDL-C), are strong risk factors for cardiovascular disease, and LDL-C reduction reduces mortality in people at risk. One of the major determinants of plasma LDL-C levels is the low density lipoprotein receptor (LDLR) that acts as a scavenger for cholesterol rich lipoprotein particles. Mutations that disrupt the function of the LDLR or lead to reduction in the number of LDLR usually result in elevated LDL-C in blood. In the current study, we identified, through whole-genome sequencing and imputation into a large fraction of the Icelandic population, four *LDLR* gene variants that affect non-HDL-C levels (that includes cholesterol in LDL and other pro-atherogenic lipoproteins) and risk of coronary artery disease (CAD). Two variants are known and two are novel. One of them, a splice region variant in intron 14 (rs72658867-A), affects normal splicing and is predicted to generate a truncated LDLR, lacking domains essential for receptor function. Despite this, rs72658867-A lowers non-HDL-C substantially and protects against CAD in the general population, demonstrating that variants that disrupt the LDLR can result in lower cholesterol levels.

## Introduction

The low-density lipoprotein receptor (LDLR) is a cell-surface receptor responsible for binding and uptake of circulating cholesterol-containing lipoprotein particles. This uptake is the primary pathway for removal of cholesterol from the circulation [1]. It is well established that high levels of low-density lipoprotein-cholesterol (LDL-C) are a key risk factor for coronary artery disease (CAD) and is a primary target for therapeutic intervention [2]. Recent studies show that non-high density lipoprotein cholesterol (non-HDL-C) is a better predictor for cardiovascular risk than LDL-C as it encompasses all cholesterol containing pro-atherogenic lipoproteins such as very low-density lipoprotein (VLDL), intermediate-density lipoprotein (IDL), chylomicron remnants (CR) as well as LDL-C [3]. LDL receptors primarily clear LDL-C from blood but they also bind VLDL-C and remnant particles [4].

The LDL receptor and its role in LDL-C regulation was discovered 40 years ago when Goldstein and Brown set out to unravel the cause of familial hypercholesterolemia (FH) [5], a severe autosomal dominant disorder characterized by high levels of LDL-C in blood and premature cardiovascular disease [6]. The most common sequence variants causing FH are loss-of-function *LDLR* mutations that disrupt the receptor function leading to reduced hepatic LDL-C clearance and elevated plasma LDL-C. So far over 1,200 rare *LDLR* mutations have been reported in FH families [7,8]. Common variants at the *LDLR* locus with modest effects on LDL-C levels and risk of coronary artery disease (CAD) in the general population have been identified through genome-wide association studies (GWAS) [9–11]. More recently GWAS studies based on whole-exome sequencing have confirmed the association between very rare *LDLR* missense and loss-of-function variants (MAF <1%) with LDL-C levels and risk of myocardial infarction (MI) [12,13]. The design of these studies, however, had little capacity to detect rare and low frequency non-coding variants at the *LDLR* locus that affect cholesterol levels and the risk of CAD and MI. High-coverage whole-genome sequencing (WGS) based GWAS in contrast have the potential to identify such variants if present.

Here we applied high-coverage WGS to a large fraction of the Icelandic population to search for *LDLR* sequence variants affecting serum levels of non-HDL-C in the general population. We found four highly significant variants each representing independent signals at the *LDLR* locus that associate with levels of non-HDL-C and risk of CAD. Two of these associations are novel and represented by non-coding variants of low frequency that lower non-HDL-C levels and protect against CAD. One of them affects splicing of the *LDLR* that is predicted to truncate the receptor.

## Results

### Identification of two low frequency non-coding variants that associate with non-HDL-C

In our study we elected to use the measurement non-HDL-C instead of LDL-C as it encompasses all potential atherogenic cholesterol containing lipoproteins including LDL-C. We examined the association of 7,351 sequence variants in a 1 Mb region centered on *LDLR* (chr19:10,559,187–11,559,187 (NCBI build36/hg18)) with non-HDL-C levels in 119,146 Icelanders. These sequence variants (SNPs and INDELs) were identified by WGS of 2,636 Icelanders and imputed, assisted by long-range phased haplotypes, into 104,220 Icelanders genotyped with Illumina SNP arrays [14]. In addition, we used genealogical information to calculate genotype probabilities for 294,212 first and second degree relatives of array genotyped individuals[15].

**Fig 1:**
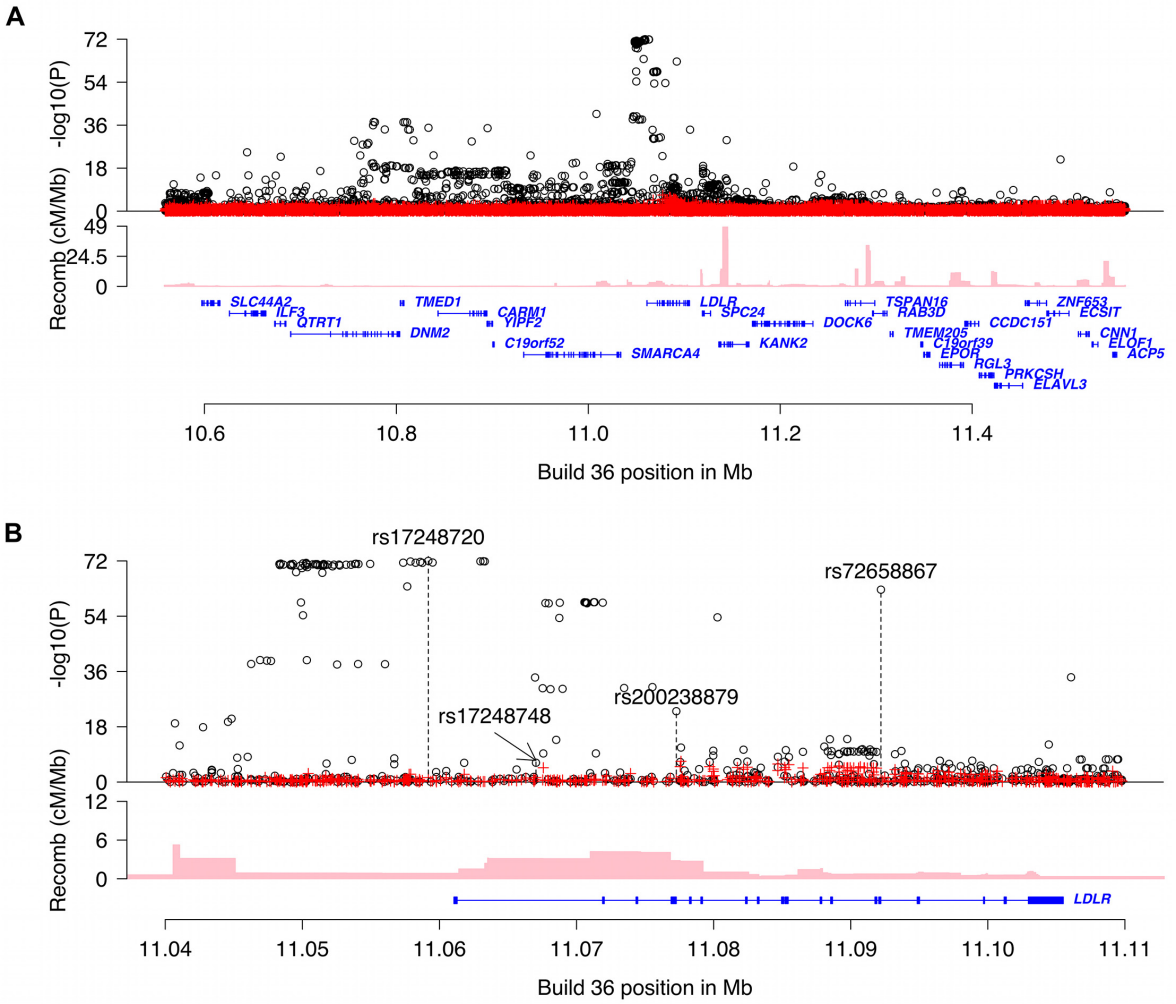
Overview of non-HDL-C associations in the region around *LDLR*. Plot **A** is a 0.8Mb overview centered on *LDLR* and plot **B** is a 70kb overview around the *LDLR* gene. Black circles show-log_10_ *P* as a function of build 36 coordinates for associations with non-HDL-C and red crosses correspond to non-HDL-C associations after adjusting for the four variants rs17248720, rs72658867, rs200238879 and rs17248748 that are indicated by vertical broken lines in plot b. Genes are shown in blue and recombination rates are reported in cM/Mb.

After performing stepwise conditional analysis we identified four highly significant variants each representing an independent signal at the *LDLR* locus (Fig 1 and Table 1 and S1 Table). Two of the variants are non-coding and low frequency and are novel with respect to association with non-HDL-C, rs72658867-A, a splice region variant at position +5 in intron 14 of *LDLR* (NM_000527.4:c.2140+5G>A, minor allelic frequency (MAF) = 2.2%), and rs17248748-T, a variant in the first intron of *LDLR* (NM_000527.4:c.68-4859C>T, MAF = 3.4%), that lower non-HDL-C by 0.44 mmol/l (*P*_*adj*_ = 2.0 × 10^−70^) and 0.13 mmol/l (*P*_*adj*_ = 5.0 × 10^−11^), respectively. The splice region variant rs72658867-A has been described in FH families, however, it has been disputed whether it is pathogenic [16–19]. The third signal is captured by a common variant rs17248720-T (NM_000527.4:c.-2038C>T, MAF = 8.8%) located at the 5’ end of *LDLR* that lowers non-HDL-C by 0.24 mmol/l (*P*_*adj*_ = 1.8× 10^−80^) and has been reported to lower LDL-C levels with similar effect as shown here and confer protection against CAD [9,20]. The fourth signal at the *LDLR* locus, is represented by a rare variant rs200238879-C (MAF = 0.06%), reported to be an Icelandic founder FH mutation [21]. This variant is located in the donor splice site of intron 4 (NM_000527.4:c.694+2T>C) and increases non-HDL-C serum levels by 1.33 mmol/l (*P*_*adj*_ = 2.2 × 10^−22^). The four variants associate with LDL-C with similar effect sizes as with non-HDL-C, the *P*-values are however slightly higher due to smaller sample size for LDL-C (S2 Table). None of the variants associate with high-density cholesterol (HDL-C) or triglycerides except rs72658867-A, that associates weakly with increased HDL-C levels (*P*_*adj*_ = 0.0035) (Table 1).

**Table 1:** Association of *LDLR* sequence variants with non-HDL-C, TG and HDL-C in Iceland.

							non-HDL-C (mmol/l)				TG (% change)				HDL (mmol/l)			
							unadjusted		adjusted		unadjusted		adjusted		unadjusted		adjusted	
Marker	chr19 pos (hg18)	A1	A2	Freq A1 ^a^ [%]	Info ^b^	*LDLR* context	*P*	β^c^	*P*	β^c^	*P*	β^d^	*P*	β^d^	*P*	β^c^	*P*	β^c^
rs17248720	11,059,187	T	C	8.8	0.99	upstream	2.6E-72	-0.23	1.8E-80	-0.24	0.50	-0.4	0.44	-0.5	0.51	0.004	0.41	0.004
rs17248748	11,067,040	T	C	3.4	0.99	intronic	5.7E-07	-0.10	5.0E-11	-0.13	0.92	0.1	0.99	0.0	0.50	0.006	0.42	0.007
rs200238879	11,077,278	C	T	0.06	0.95	splice donor	1.2E-23	1.39	2.2E-22	1.33	0.040	-12.0	0.039	-12.1	0.34	-0.054	0.36	-0.052
rs72658867	11,092,203	A	G	2.2	0.98	splice region	5.2E-63	-0.42	2.0E-70	-0.44	0.11	-1.8	0.10	-1.8	0.0042	0.029	0.0035	0.030

Association results for rs17248720, rs17248748, rs200238879 and rs72658867 with non-HDL-C, TG (triglycerides) and HDL. Association results for each variant is presented with and without adjusting for the other three variants in the table.

^a^ Freq A1 = allellic frequency for allele A1.

^b^ Info = imputation quality score.

^c^ Effect (β) in mmol/l is given with respect to the allele A1

^d^effect (β) in % change is given with respect to the allele A1.

No combination of non-HDL-C lowering alleles (minor alleles) of rs72658867-A (splice region variant), rs17248748-T (intronic) and rs17248720-T (common) occur on the same haplotype in our data (S1 Fig). The splice donor variant rs200238879-C is very rare and is weakly correlated with the other three variants (S3 Table). The non-HDL-C lowering effects of the two low frequency variants and the common variant are additive (S2 Fig and S4 Table). Homozygous carriers of each of these variants have lower non-HDL-C levels than heterozygotes, with the lowest values observed for the homozygous carriers of the splice region variant (rs72658867-A).

### Follow up of rs72658867-A and rs17248748-T association with non-HDL-C in other populations

We attempted to follow up the association of the two novel variants with non-HDL-C by direct genotyping in samples from Denmark, the Netherlands and Iran. In all three populations we replicate the association of both variants with lower non-HDL-C with similar effect sizes as in Iceland (effect of rs72658867-A on non-HDL-C is -0.41 mmol/l, *P* = 1.2 × 10^−11^ and for rs17248748-T is -0.14 mmol/l, *P* = 0.0082) (Table 2 and S5 Table). Joined with the Icelandic discovery data the combined effect on non-HDL-C for rs72658867-A is -0.44 mmol/l, *P* = 1.1 × 10^−80^ and for rs17248748-T is -0.13 mmol/l, *P* = 1.3 × 10^−12^ (Table 2 and S5 Table).

**Table 2:** Association of *LDLR* splice region variant rs72658867-A and intronic variant rs17248748-T with non-HDL-C in Denmark, Netherlands and Iran.

			non-HDL-C (mmol/l)			TG (% change)			HDL-C (mmol/l)		
Variant	Cohort (#)	Allelic freq (%)	β^e^	*P*	*P*_*het*_ ^g^	β^f^	*P*	*P*_*het*_ ^g^	β^e^	*P*	*P*_*het*_ ^g^
rs72658867-A^a^											
	Denmark (6,121)	1.18	-0.36	1.0E-04		-2.9	0.52		0.050	0.13	
	Netherlands (4,958)	0.82	-0.60	8.1E-07		-9.6	0.07		0.070	0.070	
	Iran (9,631)	0.43	-0.33	1.4E-03		-4.9	0.35		-0.001	0.97	
	Combined Replication^c^		-0.41	1.2E-11	0.15	-5.4	0.07	0.56	0.030	0.066	0.34
	Combined All^d^		-0.44	1.1E-80	0.28	-2.4	0.03	0.51	0.003	0.0006	0.49
rs17248748-T^b^											
	Denmark (6,121)	1.73	-0.16	0.039		-2.9	0.45		0.041	0.14	
	Netherlands (4,958)	1.18	-0.20	0.042		-2.1	0.65		0.036	0.26	
	Iran (9,631)	0.44	-0.04	0.71		-0.3	0.96		-0.024	0.35	
	Combined Replication^c^		-0.14	0.0082	0.46	-2.0	0.43	0.92	0.014	0.26	0.21
	Combined All^d^		-0.13	1.3E-12	0.66	-0.2	0.78	0.87	0.008	0.24	0.3

Shown are association results for rs72658867-A and rs17248748-T with non-HDL-C, TG and HDL-C in replication samples from Denmark, Netherlands and Iran.

^a^Association results are adjusted for the variants rs17248748-T and rs6511720-T (r^2^ = 0.96 with rs17248720-T in Europeans in the 1000G Phase 3 data).

^b^Association results are adjusted for the variants rs72658867-A and rs6511720-T (r^2^ = 0.96 with rs17248720-T in Europeans in the 1000G Phase 3 data).

^c^All replication samples combined for each trait.

^d^Replication samples combined with Icelandic samples, # non-HDL-C = 139,385, # TG = 100,350, # HDL = 139,753.

^e^Effect (β) in mmol/l given with respect to allele A for rs72658867 and allele T for rs17248748.

^f^Effect (β) in % change is given with respect to the allele A for rs72658867 and allele T for rs17248748.

^g^ *P*_*het*_ = *P*-value for a test of heterogeneity in the combined effect estimate.

### Association of *LDLR* variants with CAD

We tested the four *LDLR* variants for association with CAD in a sample of 33,090 cases and 236,254 controls from Iceland. All variants associate with CAD in a direction consistent with the known correlation between non-HDL-C and CAD (Table 3). The three non-HDL-C lowering variants, rs72658867-A (splice region variant), rs17248748-T (intronic) and rs17248720-T (common), all associate with a reduced risk of CAD and both rs72658867-A and rs17248720-T delay the age at diagnosis of CAD (Table 3). The rare splice donor variant that raises non-HDL-C, rs200238879-C, increases CAD risk and lowers the age at diagnosis by almost nine years (Table 3). Further, consistent with the effect on CAD, rs72658867-A and rs17248720-T both associate with increased lifespan (0.59 and 0.61 years per allele, respectively) and the rare splice donor mutation rs200238879-C associates with decreased lifespan (-6.46 years per allele) (S6 Table).

**Table 3:** Association of *LDLR* sequence variants with CAD and age at diagnosis of CAD in Iceland.

						CAD				CAD–age at diagnosis			
						unadjusted		adjusted		unadjusted		adjusted	
Marker	chr19 pos (hg18)	A1	A2	Freq A1^a^ [%]	*LDLR* context	*P*	OR^b^	*P*	OR^b^	*P*	β ^c^ [years]	*P*	β ^c^ [years]
rs17248720	11,059,187	T	C	8.8	upstream	2.1E-06	0.89	2.7E-07	0.88	0.0012	0.80	7.1E-04	0.84
rs17248748	11,067,040	T	C	3.4	intronic	0.063	0.93	0.022	0.92	0.29	0.40	0.19	0.51
rs200238879	11,077,278	C	T	0.06	splice donor	2.3E-04	2.78	2.7E-04	2.74	1.2E-04	-8.83	1.4E-04	-8.75
rs72658867	11,092,203	A	G	2.2	splice region	1.0E-07	0.77	2.7E-08	0.76	0.018	1.18	0.013	1.25

Association results for rs17248720, rs17248748, rs200238879 and rs72658867 with CAD (coronary artery disease) and age at diagnosis of CAD. Association results for each variant is presented with and without adjusting for the other three variants in the table.

^a^Freq A1 = allellic frequency of A1.

^b^OR is given with respect to allele A1.

^c^Effect (β) is given in years with respect to allele A1.

### The low frequency variants rs72658867-A and rs17248748-T are likely causative

In our WGS data the coverage at the *LDLR* locus was high (~20X) apart from a small region (50bp) in intron 1 of low coverage that was analysed separately in an independent set of individuals (n = 738) whole-genome sequenced with the Illumina PCR-free sequencing method (S3 Fig). Our dataset is thus likely to represent all sequence variants (SNPs and INDELs) at the *LDLR* locus that are present in the Icelandic population at a frequency over 0.1%, allowing for fine mapping and identification of the causative variants of the four *LDLR* signals [14]. For that purpose we explored all variants in the Icelandic dataset that correlated (*r* ^*2*^>0.8) with the four index variants in a 2 Mb window centered on each variant. The rare splice donor variant rs200238879-C is the most likely causative variant for that signal as it correlates with no other coding mutation and the mutation is known to cause abnormal splicing of the *LDLR* [21]. For the common upstream variant rs17248720 we found 56 correlates (46 variants with *r* ^*2*^>0.99) none of which are in the *LDLR* coding region (S7 Table). Three of the most strongly correlated SNPs are located in sequence motifs with strong promoter or enhancer activities in the liver cell line (HepG2) (HaploReg v3, see URLs), suggesting that any one of them could be causative.

The novel intronic variant (rs17248748-T) has no strong correlates. It is located in a sequence motif within intron 1 with strong enhancer activity in the HepG2 liver cell line and binds regulatory proteins, including c/EBPbeta known to regulate transcription of *LDLR* (HaploReg v3, see URLs), supporting a causative role of the variant [22,23]. For the splice region variant rs72658867-A we found only one correlate, rs180760728-C, an intronic variant in the *LDLR* gene (MAF = 1.98%, r^2^ = 0.89). Conditional analysis revealed that rs180760728-C does not account for the non-HDL-C association of rs72658867-A (P_*adj*_ = 0.32 for rs180760728 adjusting for rs72658867; P_*adj*_ = 8.7×10^−11^ for rs72658867-A adjusting for rs180760728-C). Furthermore, in the 1000 Genomes European ancestry Phase 3 dataset (see URLs) rs180760728-C is more weakly correlated with rs72658867-A (*r* ^*2*^ = 0.49) and rs180760728 is the only marker with *r* ^*2*^>0.3 with rs72658867-A, indicating that rs72658867-A is likely the causative variant for this signal. The replication of the non-HDL-C associations of rs72658867-A and rs17248748-T with similar effect sizes in the three distinct populations further supports a causative role for these variants.

### The splice region variant rs72658867-A associates with higher *LDLR* mRNA expression in white blood cells

**Fig 2:**
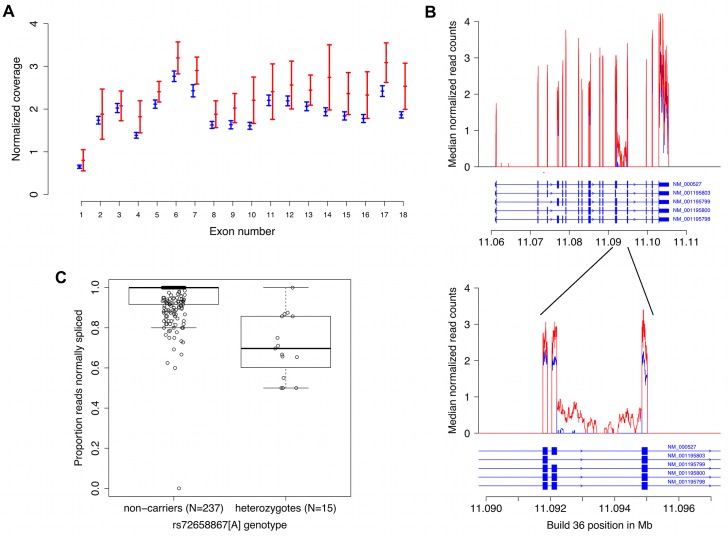
RNA sequencing data from blood demonstrates increased expression and abnormal splicing characterized by intron 14 retention in carriers of the splice region variant rs72658867-A. **A.** Normalized average *LDLR* exon coverage for non-carriers (*N* = 238, in blue) and heterozygotes (*N* = 15, in red) of rs72658867-A demonstrates increased expression of *LDLR* transcripts in heterozygotes by ~22%, *P* = 0.0075. The X-axis is the exon number corresponding to RefSeq transcript NM_000527 for *LDLR*. The Y-axis shows the median normalized coverage (normalized for each individual to the total number of aligned reads). The error bars are based on the median absolute deviation within each group and is calculated separately for each exon. **B.** Using the same samples as in a) preferential intron 14 retention is observed in heterozygous carriers of rs72658867-A (shown in red). The X-axis is the genomic position in Mb (hg18/Build36). The Y-axis is the median count of normalized reads as in a). The structure of all *LDLR* RefSeq transcript variants is shown. The upper panel shows the full length gene whereas the lower panel shows the exons 13, 14 and 15 and the intron retention in intron 14. **C.** Quantitation of the proportion of transcripts with intron 14 retention in heterozygotes. The Y-axis corresponds to the proportion of RNA sequencing reads that are spliced from exon 14 to exon 15 (correctly spliced) out of the total number of reads that cover the last base of exon 14 (individuals that do not have coverage at this position are omitted). Median proportion: 1.00 (non-carriers); 0.70 (heterozygotes). Mean proportion: 0.95 (non-carriers); 0.71 (heterozygotes). Mann-Whitney test for location shift gives *P* = 6.0×10^−9^.

To further characterize the two novel non-HDL-C signals at the *LDLR* locus we analysed the effect of rs72658867 and rs17248748 on *LDLR* mRNA expression in a microarray mRNA expression dataset for white blood cells (1,001 individuals) and adipose tissue (667 individuals). The non-HDL-C lowering allele of the splice region variant rs72658867 associates with increased *LDLR* mRNA expression in blood (~22% increase, *P* = 1.2 × 10^−11^) (S4 Fig) and no other variant in a ~1Mb region centered on *LDLR* correlated more strongly with *LDLR* expression than rs72658867 (S8 Table). These findings were replicated in an independent RNA-sequencing dataset from blood (252 individuals) where similar increase was detected in carriers (*P* = 0.0075) (Fig 2A). Using that dataset we also performed allele specific analysis of heterozygous carriers and non-carriers and show that the chromosomes carrying rs72658867-A have greater expression than the chromosomes carrying the reference allele (S9 Table and S5 Fig). In heterozygous carriers of rs72658867-A about 60% of the transcripts are derived from the mutated chromosome compared to a baseline proportion of 0.52 in non-carriers. In the adipose tissue microarray dataset the correlation with *LDLR* expression is in the same direction but much weaker (*P* = 0.020, S4 Fig). No significant correlation with *LDLR* expression in blood or adipose tissue was found for the intronic rs17248748 variant (*P* = 0.40 and *P* = 0.10, respectively).

### The splice region variant rs72658867-A disrupts *LDLR* splicing and is predicted to truncate the receptor

The splice region variant rs72658867 is located at position 5 of intron 14, a position that is conserved and could potentially affect splicing. To investigate this, we analysed the mRNA sequence data from blood and observed abnormal splicing in rs72658867-A carriers, characterized by retention of intron 14 (i.e. transcription through intron 14 in the *LDLR* transcripts) (Fig 2B). When looking at the proportion of RNA sequencing reads that are spliced from exon 14 to exon 15 (correctly spliced) out of the total number of reads that cover the last base of exon 14 we observed a mean proportion of 0.95 in non-carriers (n = 238) compared to 0.71 in heterozygous carriers (n = 15) (Mann-Whitney test for location shift *P* = 6.0×10^−9^). This indicates that approximately 30% of the transcripts in heterozygous carriers are abnormally spliced (Fig 2C). Analysis of blood RNA sequence data from homozygous carriers of rs72658867- A (n = 3) demonstrated that about half of the *LDLR* transcripts are characterized by intron retention (S10 Table). Together these data from the hetero- and homozygous carriers indicate that from chromosomes carrying rs72658867-A, about half of the transcripts are normally spliced and half of them are abnormal. This also indicates that even though the total amount of *LDLR* transcripts is increased by 22% in heterozygotes (Figs 2A and S4), the estimated amount of normally spliced *LDLR* transcripts will not exceed 90% of normal levels. This was confirmed by RT-PCR analysis of *LDLR* mRNA from blood that showed similar levels of wild type *LDLR* mRNA in heterozygous rs72658867-A/G carriers (n = 20) and rs72658867-G/G non-carriers (n = 343), (Mann-Whitney test: *P* = 0.87) (S6 Fig). The non-HDL-C lowering effect of rs72658867-A is thus not mediated by a net increase in the wild type *LDLR* transcripts.

The retention of intron 14 alters the LDLR reading frame after amino acid position 713 (end of exon 14, NP000518:p.Thr713fsTer33) such that 33 amino acids are added until a premature stop codon is reached. It is unlikely that the introduction of the premature stop codon renders the transcript susceptible to nonsense-mediated decay as a high fraction of the *LDLR* transcripts in heterozygotes and homozygotes are characterized by retention of intron 14 (Fig 2C and S10 Table). The abnormally spliced mRNA is predicted to produce a truncated LDLR lacking the O-linked glycan region and the transmembrane and cytoplasmic domains (S7 Fig). The transmembrane domain anchors the LDLR in the lipid bilayer and endocytosis and intracellular transport of the LDLR are regulated via its cytoplasmic domain [24] but the role of the O-linked glycan region is unclear[25].

## Discussion

We have identified, by high coverage whole-genome sequencing and subsequent imputation into a large fraction of the Icelandic population, four independent sequence variants at the *LDLR* locus that associate with levels of non-HDL-C and risk of CAD in the general population. Two of them are of low frequency and novel with respect to non-HDL-C association: a splice region variant (rs72658867-A, c.2140+5G>A) and an intronic variant (rs17248748-T). Both variants associate with lowering of non-HDL-C and protection against CAD. We show that the splice region variant causes retention of intron 14 altering the LDLR reading frame after amino acid position 713 such that 33 amino acids are added until a premature stop codon is reached. This splicing defect affects about half of the transcripts generated from the chromosome carrying the variant. The same variant also increases mRNA expression of *LDLR* that includes both normally and abnormally spliced transcripts and this increase seems to be driven by the chromosome carrying rs72658867-A. This study highlights the importance of including non-coding variants, in all segments of the frequency spectrum (common, low frequency and rare), in GWAS.

Although the abnormal transcript is predicted to translate into a truncated LDLR lacking domains essential for receptor function, the splice region variant associates with a strong non-HDL-C lowering effect and protection against CAD in the general population. These data contrast *LDLR* truncating mutations that lead to an increase in non-HDL-C because of reduced function of the LDLR [24–26]. The evidence for the non-HDL-C lowering effect of rs72658867-A is further strengthened by replication of the effect in three additional populations. Since the splice region mutation also increases expression of the *LDLR* mRNA, the non-HDL-C lowering could be mediated by an increase above normal levels in wild type *LDLR* transcripts. We, however, demonstrate that the wild type mRNA levels are comparable in heterozygous rs72658867-A carriers and non-carriers. Furthermore, others have shown that in a lymphoblastoid cell line generated from a heterozygous carrier of rs72658867-A, the membrane bound LDLR levels and internalization of LDL are similar to that of cell lines that do not carry *LDLR* mutations [27]. The non-HDL-C lowering effect of rs72658867-A is thus not mediated by a net increase in the wild type *LDLR* transcripts. *LDLR* mutations have been described that cause truncation of the receptor at similar location as rs72658867-A is predicted to do [24–26], however, these mutations are different from rs72658867-A in that they appear to lead to reduction in wild type transcripts. In contrast to rs72658867-A, these mutations have been linked to FH and an increase in non-HDL-C. Perhaps the combination of a truncated receptor and normal wild type levels of the LDLR mediate the non-HDL-C lowering effect of rs72658867-A.

The observed effect of rs72658867-A on *LDLR* splicing can be attributed to its spatial relation to the site of splicing (c.2140+5G>A). Allele specific analysis of mRNA sequence data indicates that the increase in *LDLR* transcripts in rs72658867-A carriers is derived from the chromosome carrying rs72658867-A. The *LDLR* splicing defect and increased *LDLR* mRNA expression are thus likely both mediated by the splice region variant rs72658867-A itself since in Iceland no other variant than rs72658867-A can fully explain the association with non-HDL-C and in the 1000G European ancestry data (Phase 3 dataset, see URL) no variant is correlated with *r* ^*2*^>0.5 with rs72658867-A. It is however, unlikely that the effect of rs72658867-A on *LDLR* expression is mediated by the splicing defect itself. Based on ENCODE data, rs72658867 overlaps a RNA polymerase II binding site in number of different cell lines which may possibly reflect an enhancer site that could mediate altered *LDLR* expression.

In conclusion we have identified two non-coding low frequency variants in the *LDLR* gene that associate with lower non-HDL-C and protection against CAD. One of them, the splice region variant rs72658867-A, affects splicing and introduces a premature stop codon that is expected to produce a truncated LDLR lacking the O-linked glycan region and the transmembrane and cytoplasmic domains, domains that are both essential for function of the receptor. The same mutation increases transcription of the *LDLR*, albeit the normal wild type transcript levels do not exceed levels detected in non-carriers. These data contrast the effects of other reported LDLR truncating mutations that increase LDL-C levels and the risk of CAD. Further functional studies are warranted to gain better understanding of the biology of the splice region variant rs72658867-A.

## Materials and Methods

### Ethics statement

The study was approved by The National Bioethics Committee in Iceland (Approval no. 07–085, with amendments), and the Data Protection Authority in Iceland (Approval no. 2007060474ÞS/—, with amendments). All donors of biological samples gave informed written consent.

### Study populations

#### Iceland

We obtained blood lipid measurements (non-high-density lipoprotein (non-HDL-C), low-density lipoprotein (LDL-C) and high-density lipoprotein (HDL-C) cholesterol as well as triglycerides (TG)) from three of the largest laboratories in Iceland: (i) Landspítali—The National University Hospital of Iceland (LUH), Reykjavík, Iceland (measurements performed between the years 1993 and 2012, hospitalized and ambulatory patients); (ii) The Laboratory in Mjódd (RAM), Reykjavík, Iceland (measurements performed between 2004 and 2012, ambulatory patients); and (iii) Akureyri Hospital, The Regional Hospital in North Iceland, Akureyri, Iceland (performed between 2004 and 2010, hospitalized and ambulatory patients). Measurements performed at the Icelandic Heart Association at the time of recruitment for deCODE´s studies between the years 1999 and 2004 were also included. The participants had a median of 2 (1–86) and geometric average of 2.5 non-HDL-C measurements. The mean non-HDL-C value was 4.00 mmol/l (SD = 1.18). Lipid levels were adjusted for sex, year of birth and age at measurement, lipid lowering medication and measurement site, using the average of multiple measurements for an individual, and then normalized to a standard normal distribution using quantile normalization. To obtain effect estimates in mmol/L the estimates from the regression analysis were multiplied by the estimated standard deviation of lipid level in the population. Given their approximately log-normal distribution, triglyceride levels were log-transformed before adjustment and the corresponding effect estimates are presented as percentage change instead of units of mmol/L. A total of 119,146 individuals with non-HDL-C were included in the study, where 69,277 were chip-typed and directly imputed; the remaining 49,869 were first and second degree relatives of chip-typed individuals and had their genotypes inferred based on genealogy[15]. Non-HDL-C is obtained by subtracting HDL-C from total cholesterol and gives a measure of the amount of cholesterol carried within all atherogenic lipoprotein particles (VLDL, IDL and LDL).

Coronary artery disease (CAD) was defined as: (a) individuals in the MONICA registry who suffered myocardial infarction (MI) before the age of 75 in Iceland between 1981 and 2002 and satisfied the MONICA criteria[28], (b) subjects with CAD discharge diagnoses (ICD 9 codes 410.*, 411.*, 412.*, 414.* or ICD 10 codes I20.0, I21.*, I22.*. I23.*, I24.*, I25.*) from LUH, (c) subjects diagnosed with significant angiographic CAD (see below) identified from a nationwide clinical registry of coronary angiography and percutaneous coronary interventions (PCI) at LUH between the years 1987 and 2012, (d) subjects undergoing coronary artery bypass grafting (CABG) procedures at LUH between the years 2002 and 2011 or (e) cause of death or contributing cause of death listed as MI or CAD (ICD 9 or 10 codes) on death registries between the years 1996 and 2012. Coronary angiograms in the nationwide registry were evaluated by an interventional cardiologist. Patients were considered to have significant angiographic CAD if one or more of the three major epicardial coronary vessels or the left main coronary artery was found to have at least 50% diameter stenosis by visual estimation. A total of 33,090 CAD cases were used in the study. Of those 14,640 were chip-typed and imputed and the remaining 18,450 were first and second degree relatives of chip-typed individuals and had their genotypes inferred based on genealogy. The 236,254 Icelandic controls were study participants from various deCODE genetics programs without known CAD. The lifespan variable was based on 62,558 individuals that were born after 1890 and lived to be at least 50 years old. Personal identities of the patients and biological samples were encrypted by a third party system provided by the Icelandic Data Protection Authority.

#### Denmark

The Danish samples are part of the randomised population-based intervention study (Inter99) which has been described in details elsewhere[29]. Danish study participants gave informed consent for use of their biological samples for genetic studies. The current research protocol was approved by The Danish National Ethical Committee on Health Research and is in accordance with the ethical scientific principles of the Helsinki Declaration II.

#### The Netherlands

Subjects from the Netherlands were recruited within the ‘Nijmegen Biomedical Study’. The details of this study were reported previously [30]. Briefly, this is a population-based survey conducted by the Department for Health Evidence and the Department of Clinical Chemistry of the Radboud. Age- and sex-stratified randomly selected adult inhabitants of Nijmegen were invited to participate and to donate a blood sample for DNA isolation and biochemical studies. The Nijmegen Biomedical Study was approved by the Institutional Review Board of the Radboud University medical center.

#### Iran

The Iranian samples are part of the Tehran Lipid and Glucose Study (TLGS) cohort. The TLGS design has been described in detail previously [31]. The study has been ongoing for 20 years and 15,005 residents of District 13 of Tehran have been enrolled. For the purpose of this study we included only individuals ≥18 years of age. The study was approved by the National Research Council of the Islamic Republic of Iran.

Lipid values for the Danish, Dutch and Iranian samples were adjusted and standardized as in Iceland.

### Genotyping

#### Illumina SNP chip-genotyping

Icelandic chip-typed samples were assayed using the Illumina HumanHap300, HumanCNV370, HumanHap610, HumanHap1M, HumanHap660, Omni-1, Omni 2.5 or Omni Express bead chips at deCODE genetics. SNPs were excluded if they had (i) yield <95%, (ii) MAF <1% in the population or (iii) significant deviation from Hardy–Weinberg equilibrium in the controls (P <0.001), (iv) if they produced an excessive inheritance error rate (over 0.001) or (v) if there was substantial difference in allele frequency between chip types (from just a single chip if that resolved all differences, but from all chips otherwise). All samples with a call rate below 97% were excluded from the analysis.

#### Single track assay SNP genotyping

Single SNP genotyping was carried out applying the Centaurus (Nanogen) platform[32].

#### Whole-genome sequencing

Whole-genome sequencing was performed for 2,636 individuals selected for various conditions. All individuals were sequenced at a minimum depth of 10x (average 22x). Sample preparation, DNA sequencing and alignment were recently described in detail [15]. Briefly, paired-end libraries for sequencing were prepared according to the manufacturer’s instructions (Illumina, TruSeqTM), sequencing by synthesis was performed on Illumina GAIIx and/or HiSeq 2000 instruments and reads were aligned to NCBI Build 36 of the human reference sequence using Burrows-Wheeler Aligner (BWA) 0.5.9[33].

### Long-range phasing and genotype imputation

Long-range phasing of all chip-genotyped individuals was performed with methods previously described[34,35]. For the HumanHap series of chips, 304,937 SNPs were used for long-range phasing, whereas for the Omni series of chips 564,196 SNPs were included. The final set of SNPs used for long-range phasing was composed of 707,525 SNPs. A detailed description of imputation methods used for the Icelandic population was recently published[15]. In brief, SNPs and INDELs identified through sequencing were imputed into 104,220 chip-genotyped and long-range phased Icelanders. Approximately 28.3 million SNPs and small INDEL variants were imputed based on this set of individuals. The imputation quality score for the four highly significant *LDLR* sequence variants, rs17248720-T, rs17248748-T, rs200238879-C and rs72658867-A was 0.99, 0.99, 0.95 and 0.98, respectively (Table 1).

### Association analysis

A generalized form of linear regression that accounts for relatedness between individuals was used to test for the association of quantitative traits with sequence variants[36]. Conditional analysis was performed by including the sequence variant being conditioned on as a covariate in the model under the null and the alternative in the generalized linear regression. Stepwise forward selection was used starting with sequence variants as a covariate in the model then adjusting for the most significant sequence variant and repeating that process until no variant remained significant in the region. Logistic regression was used to test for association between sequence variants and disease (CAD), treating disease status as the response and expected genotype counts from imputation or allele counts from direct genotyping as covariates. Other available individual characteristics that correlate with disease status were also included in the model as nuisance variables. These characteristics were: sex, county of birth, current age or age at death (first- and second-order terms included), blood sample availability for the individual and an indicator function for the overlap of the lifetime of the individual with the timespan of phenotype collection. Testing was performed using the likelihood ratio statistic.

### Correction for relatedness of Icelandic subjects and population stratification

Individuals in both the Icelandic case and control groups are related, causing the χ^2^ test statistic to have a mean >1 and median >0.675. We estimated the inflation factor λ_g_ based on a subset of about 300,000 common variants and the *P*-values were adjusted by dividing the corresponding χ^2^ values by this factor to adjust for both relatedness and potential population stratification[37]. Genomic control correction factors: non-HDL-C: 1.36, triglycerides: 1.40, HDL-C: 1.575, CAD: 1.71, CAD age of onset: 1.41, lifespan: 1.49.

### Genotype imputation information

The informativeness of genotype imputation was estimated by the ratio of the variance of imputed expected allele counts and the variance of the actual allele counts:

Var[E(θ|chipdata)]Var(θ),

where θ∈{0,1} is the allele count. Var[E(θ|chipdata)] was estimated by the observed variance of the imputed expected counts and Var(θ) was estimated by *p*(1 − *p*), where *p* is the allele frequency. Sequence variants with imputation information below 0.8 were excluded from the analysis.

### Gene and variant annotation

Sequence variants were annotated with information from Ensembl release 70 using Variant Effect Predictor (VEP) version 2.8[38]. Variants annotated as having high impact include loss-of-function variants, i.e. stop-gained variants, frameshift indels and essential splice variants, and moderate impact variants include missense, inframe indels, and splice region variants.

### Gene Expression Microarrays

Samples of RNA from human peripheral blood were hybridized to Agilent Technologies Human 25K microarrays as described previously[39]. We quantified expression changes between two samples as the mean logarithm (log10) expression ratio (MLR) compared to a reference pool RNA sample. In comparing expression levels between groups of individuals with different genotypes, we denoted the expression level for each genotype as 10 (average MLR), where the MLR is averaged over individuals with the particular genotype. We determined s.e.m. and significance by regressing the MLR values against the number of risk alleles carried. We took into account the effects of age, gender and differential cell type count in blood as explanatory variables in the regression. *P*-values were adjusted for familial relatedness of the individuals by simulation.

### RNA-analysis

#### Preparation of Poly-A cDNA sequencing libraries

The quality and quantity of isolated total RNA samples was assessed using the Total RNA 6000 Nano chip for the Agilent 2100 Bioanalyzer. cDNA libraries derived from Poly-A mRNA were generated using Illumina´s TruSeq RNA Sample Prep Kit. Briefly, Poly-A mRNA was isolated from total RNA samples (1–4 μg input) using hybridizaton to Poly-T beads. The Poly-A mRNA was fragmented at 94°C, and first-strand cDNA was prepared using random hexamers and the SuperScript II reverse transcriptase (Invitrogen). Following second-strand cDNA synthesis, end repair, addition of a single A base, adaptor ligation, AMPure bead purification, and PCR amplification, the resulting cDNA was measured on a Bioanalyzer using the DNA 1000 Lab Chip.

#### Sequencing

Samples were clustered on to flowcells using Illumina´s cBot and the TruSeq PE cluster kits v2, respectively. Paired-end sequencing (2x76 cycles) was performed with either GAIIx instruments using the TruSeq SBS kits v5 from Illumina or HiSeq 2000 instruments using TruSeq v3 flowcells/SBS kits. In some instances the readlength was 2x50 or 2x101 cycles. Approximately 125–175 million forward reads (250–350 M total reads) were sequenced per sample.

#### Read alignment

RNA sequencing reads were aligned to Homo sapiens Build 36 with TopHat[40] version 1.4.1 with a supplied set of known transcripts in GTF format (RefSeq hg18; Homo sapiens, NCBI, build36.3). TopHat was configured such that it attempts first to align reads to the provided transcriptome then, for reads that do not map fully to the transcriptome, it attempts to map them onto the genome. Read mapping statistics used for read count normalization were calculated using the CollectRnaSeqMetrics tool in Picard version 1.79 (see URLs).

#### RNA-seq data analysis

For each sample, the normalized read count was determined for each bp location as # reads covering the base / the total # of aligned reads in the individual in billions. Then the median normalized read count for each genotype group was determined for each bp location and plotted graphically. In order to quantify the levels of expression in different regions, the median of normalized read counts over the genomic segment in question was determined for each individual. We then did a logarithmic transformation of the normalized read counts and standardized them so that the response variable had a mean 0 and standard deviation of 1.

#### Allele specific expression analysis of LDLR for rs72658867 heterozygous carriers

To analyze allele specific expression for heterozygous carriers of rs72658867-A we looked at base counts in the RNA-Seq data at positions of synonymous variants in heterozygous state (see S8 Table for list of the allele specific synonymous variants tested). All the synonymous variants we considered had D’≥0.99 with the splice region variant rs72658867-A. Using the phasing information for the imputed genotypes of the samples, we calculated the proportion of read bases at the positions of the synonymous variants, where the proportion was #ALT bases/#total bases if the alternative allele (ALT) of the synonymous variant was positively correlated with rs72658867-A and #REF bases/#total bases if the reference allele (REF) of the synonymous variant was negatively correlated with rs72658867-A. The median proportions over all synonymous variants for rs72658867 GG and GA carriers were 0.52 and 0.61, respectively (*P* = 0.0016, Mann-Whitney test, making a simplifying assumption of independence of proportions); the median proportion for rs72658867 GG being greater than 0.50 reflects a reference bias in the mapping of RNA-Seq reads.

#### Quantitative RT-PCR

Quantitative RT-PCR of the LDLR cDNA, specific for the correct removal of intron 14, was performed with an assay from the Roch Universal probe library. This was run on an ABI 7900HT Real-time PCR system according to standard protocol (forward primer: ccactcgcccaagtttacc, reverse primer: gcagcctcagcctctgtg, probe #17).

### URLs

Picard: http://broadinstitute.github.io/picard/command-line-overview.html#CollectRnaSeqMetrics

HaploReg v3 (accessed May 2015):

http://www.broadinstitute.org/mammals/haploreg/haploreg_v3.php

1000genomes Phase 3 (May 2013): ftp://ftp.1000genomes.ebi.ac.uk/vol1/ftp/release/20130502/

## Supporting Information

S1 Fig Haplotype counts based on phased genotypes for n = 2,577 sequenced individuals. We denote the five observed haplotypes by H0 (wild type; major allele for all four variants), H1 (carrying minor allele of upstream variant rs17248720-T), H2 (carrying minor allele of splice region variant rs72658867-A), H3 (carrying minor allele of intronic variant rs17248748-T) and H4 (carrying minor allele of splice donor variant rs200238879-C). Haplotypes are shown schematically where the chromosome is drawn as a line and mutations are represented by the symbol ‘×’. Allele frequencies are based on imputed genotypes.
(PDF)

S2 Fig Non-HDL-C levels for individuals with phased and imputed genotypes for the three non-HDL-C lowering variants rs17248720-T (upstream, MAF = 8.8%), rs72658867-A (splice region, MAF = 2.2%) and rs17248748-T (intronic, MAF = 3.4%). No combination of the minor alleles of these three variants occur on the same haplotype. We denote the four possible haplotypes by H0 (major allele for all three variants), H1 (carrying minor allele of upstream variant rs17248720-T), H2 (carrying minor allele of splice region variant rs72658867-A) and H3 (carrying minor allele of intronic variant rs17248748-T).
(PDF)

S3 Fig Total sequencing read coverage per base over *LDLR* for n = 2,636 whole-genome-sequenced individuals. The structure of all *LDLR* RefSeq transcripts variants are shown in panel below.
(PDF)

S4 Fig Increased expression of the *LDLR* transcript containing the rs72658867-A (c.2140+5G>A) mutation. Expression of *LDLR* in (a) blood and (b) adipose tissue in rs72658867 non-carriers (GG) and carriers (AG). *P*-values are from the regression of the average log expression ratio on the carrier status, adjusting for age and sex, and differential counts for blood.
(PDF)

S5 Fig Allele specific expression analysis of *LDLR* for rs72658867 heterozygous carriers using base counts for synonymous variants in heterozygous state (see S8 Table for the variants used). Shown is the proportion of read bases for the synonymous variants in heterozygous state for rs72658867 GG and GA carriers, where the numerator is base count for the allele correlated with rs72658867[A]. Median proportion for rs72658867 GG and GA carriers are 0.52 and 0.61, respectively (*P* = 0.0016, Mann-Whitney test). Broken line corresponds to proportion of 0.50; the median proportion for the non-carriers being higher reflects reference mapping bias of RNA-Seq reads.
(PDF)

S6 Fig Quantitative RT-PCR of the *LDLR* wild type cDNA (amplifies only cDNA with correctly spliced intron 14). Boxplots show *LDLR* expression relative to beta-actin for rs72658867 in non-carriers (median: 0.062) and heterozygotes (median: 0.061). No significant difference in expression was found for the two groups (Mann-Whitney test: *P* = 0.87).
(PDF)

S7 Fig Expected consequence of the splice region variant rs72658867-A (c.2140+5G>A) on the LDL receptor. A schematic diagram of the domain structure of the LDLR protein is shown at the top (domains deleted from the truncated receptor are filled). The wild type nucleotide and amino acid sequence for the end of exon 14 and start of exon 15 is shown in the middle. At the bottom: the nucleotide sequence of the abnormally spliced RNA (in red intron 14 retention, in bold is the splice site mutation, c.2140+5G>A) and the amino acid sequence of the truncated receptor (in blue 33 novel amino acids).
(PDF)

S1 Table Associations of sequence variants in a 1Mb region centered on *LDLR* (chr19:10,599,187–11,559,187 (NCBI build36/hg18)) with non-HDL-C levels in 119,146 Icelanders. Shown are results with *P*<0.05. Column descriptions: Amin: Minor allele; Amaj: major allele; MAF: minor allele frequency as percentage; Info: imputation information; coding gene, coding effect and coding change correspond to gene symbol, consequence of mutation and HGVSp annotation, respectively (based on Ensembl' s Variant Effect Predictor (VEP)).
(XLSX)

S2 Table Association of *LDLR* sequence variants with non-HDL-C and LDL-C in Iceland. Association results for rs17248720, rs17248748, rs200238879 and rs72658867 with non-HDL-C and LDL-C. Association results for each variant is presented with and without adjusting for the other three variants in the table. ^a^ Freq A1 = allellic frequency for allele A1. ^b^ Info = imputation quality score. ^c^ Effect (β) in mmol/l is given with respect to the allele A1.
(DOCX)

S3 Table D' and r^2^ between the four non-HDL-C associated variants at LDLR. f_1_ and f_2_ are the allele frequencies for variants 1 and 2, respectively. β_1_ and β_2_ correspond to (adjusted) effects on non-HDL-C with respect to variants 1 and 2. Sign of correlation is with respect to the alleles of the variants. Calculations are based on 104,202 imputed Icelandic individuals that have phased genotypes.
(DOCX)

S4 Table Non-HDL-C levels for individuals with phased and imputed genotypes for the three non-HDL-C lowering variants rs17248720-T, rs72658867-A and rs17248748-T. *We denote the four possible haplotypes by H0 (major allele for all three variants), H1 (carrying minor allele of upstream variant rs17248720-T), H2 (carrying minor allele of splice region variant rs72658867-A) and H3 (carrying minor allele of intronic variant rs17248748-T). The notation Hn|Hm stands for the individual that carries haplotype Hn on one chromosome and Hm on the other (n,m = 0,1,2,3).
(DOCX)

S5 Table Association of *LDLR* sequence variants with non-HDL-C, TG and HDL-C in Iceland, Denmark, Netherlands and Iran. ^a^Results presented are for rs6511720-T (r^2^ = 0.96 with rs17248720-T in Europeans in the 1000G Phase 3 data). ^b^All replication samples combined for each trait. ^c^Replication samples combined with Icelandic samples, # non-HDL-C = 139,385, # TG = 100,350, # HDL = 139,753. ^d^ = effect β in units of standardized distribution (SD) given with respect to allele T for rs17248720, allele A for rs72658867 and allele T for rs17248748. ^e^effect in mmol/l is given with respect to allele T for rs17248720, allele A for rs72658867 and allele T for rs17248748. ^f^effect in % change is given with respect to allele T for rs17248720, allele A for rs72658867 and allele T for rs17248748. ^g^ *P*-value for a test of heterogeneity in the combined effect estimate.
(XLSX)

S6 Table Association of *LDLR* sequence variants with lifespan. Association results for rs17248720, rs17248748, rs200238879 and rs72658867 with lifespan. Lifespan corresponds to individuals born after 1890 that lived to be 50 years old. Effect (β) in years is given with respect to the allele A1. Each variant is adjusted for the other three variants in the table.
(DOCX)

S7 Table Variants that have r^2^>0.8 with the upstream variant rs17248720-T in a 2Mb window around it. ^a^Effect (β) is given with respect to the allele A1. ^b^The index variant rs17248720 is included in the table.
(DOCX)

S8 Table Markers with eQTL *P*<1e-4 in white blood cells for *LDLR* probe (NM_000527) in the *LDLR* region. ^a^β given with respect to A1, ^b^the two top eQTL variants rs72658867 and rs180760728 are correlated (r^2^ = 0.89). The non-HDL-C association for the second best eQTL variant rs180760728 is not significant after adjusting for rs72658867 (*P*_adj_ = 0.32, β_adj_ = 0.32). The non-HDL-C association for rs72658867 remains significant once adjusted for the second best eQTL variant (*P*_*adj*_ = 8.7e-11, β_adj_ = -0.41).
(DOCX)

S9 Table Allele specific expression analysis for rs72658867-A heterozygous carriers and non-carriers using base counts at synonymous variants in heterozygous state. Allele specific expression was evaluated with five different allele specific markers. r^2^, D’ and sign correspond to LD calculations for rs72658867-A and alternative allele (ALT) for the allele specific markers; if sign = 1, then the allele rs72658867-A is correlated with the ALT allele of the marker; if sign = -1, then the allele rs72658867-A is correlated with the reference allele (REF) of the marker. Mean, std. dev and median correspond to the proportion of read bases for the allele specific marker (if sign = 1, proportion = #ALT bases/#total bases; if sign = -1, proportion = #REF bases/#total bases). Δmedian: median rs72658867 GA–median rs72658867 GG. (Note: The median for the rs72658867 heterozygotes is always higher than the median for the non-carriers.) *P* corresponds to the significance level for testing difference in the read base proportions for the two groups (rs72658867 GA and rs72658867 GG) for the allele specific markers, using a two-sided Mann-Whitney test.
(DOCX)

S10 Table Analysis of splicing of exons 14 and 15 in LDLR based on RNA-seq from three blood donors who are homozygous for the splice region variant rs72658867-A. The mean ratio of coverages for intron 14 vs. exon 14 is 0.21, indicating an intron retention. To quantitate the proportion of transcripts with intron 14 retention we consider the proportion of RNA sequencing reads that are spliced from exon 14 to exon 15 (correctly spliced) out of the total number of reads that cover the last base of exon 14; the mean proportion of reads spliced is 0.49, indicating that about half of the LDLR transcripts are incorrectly spliced.
(DOCX)
