# Supplementary material for: A Splice Region Variant in LDLR Lowers Non-high Density Lipoprotein Cholesterol and Protects against Coronary Artery Disease
Source: PLoS Genet. 2015 Sep 1;11(9):e1005379. doi: 10.1371/journal.pgen.1005379 (PMC4556698; doi:10.1371/journal.pgen.1005379)
Supplement: S1 Fig — We denote the five observed haplotypes by H0 (wild type; major allele for all four variants), H1 (carrying minor allele of upstream variant rs17248720-T), H2 (carrying minor allele of splice region variant rs72658867-A), H3 (carrying minor allele of intronic variant rs17248748-T) and H4 (carrying minor allele of splice donor variant rs200238879-C). Haplotypes are shown schematically where the chromosome is drawn as a line and mutations are represented by the symbol ‘×’. Allele frequencies are based on imputed genotypes. (PDF) [file pgen.1005379.s001.pdf]

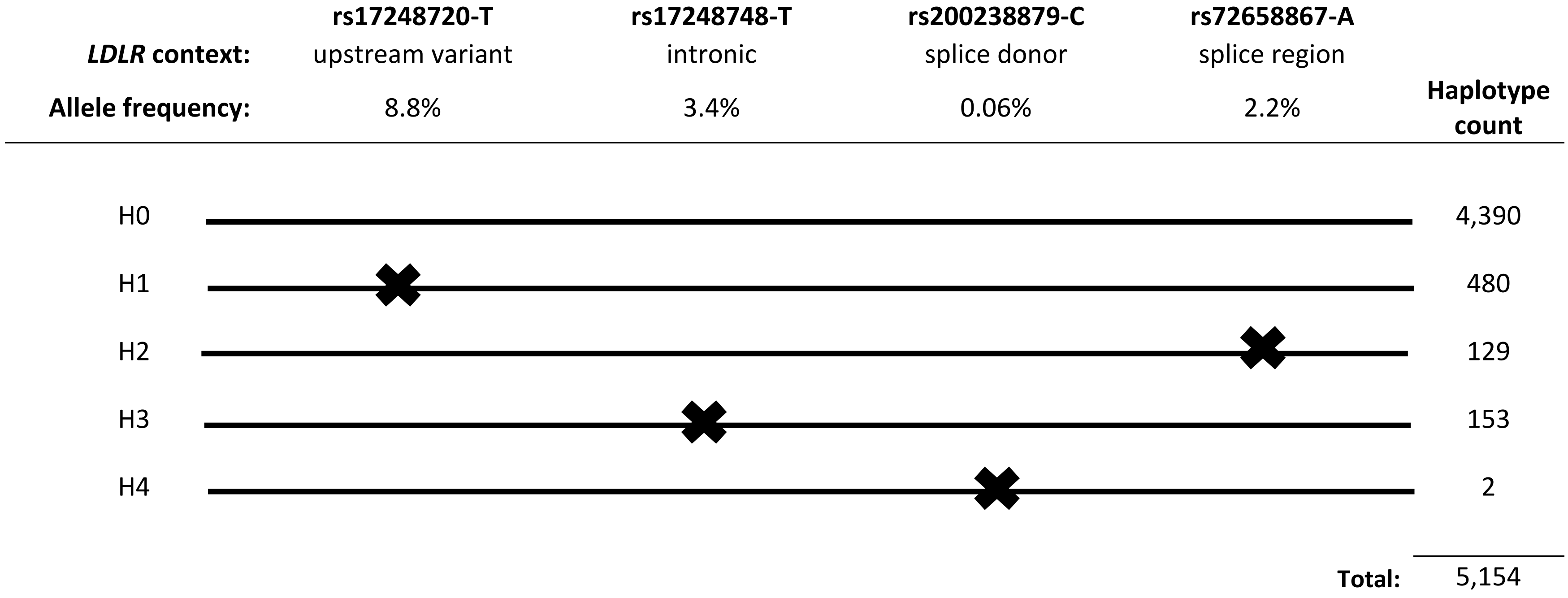
rs17248720-T
rs17248748-T
rs200238879-C
rs72658867-A
LDLR context:
upstream variant
intronic
splice donor
splice region
Allele frequency:
8.8%
3.4%
0.06%
2.2%
Haplotype count
H0
4,390
H1
480
H2
129
H3
153
H4
2
Total:
5,154
