# Supplementary figures and images for: A Splice Region Variant in LDLR Lowers Non-high Density Lipoprotein Cholesterol and Protects against Coronary Artery Disease
Source: PLoS Genet. 2015 Sep 1;11(9):e1005379. doi: 10.1371/journal.pgen.1005379 (PMC4556698; doi:10.1371/journal.pgen.1005379)

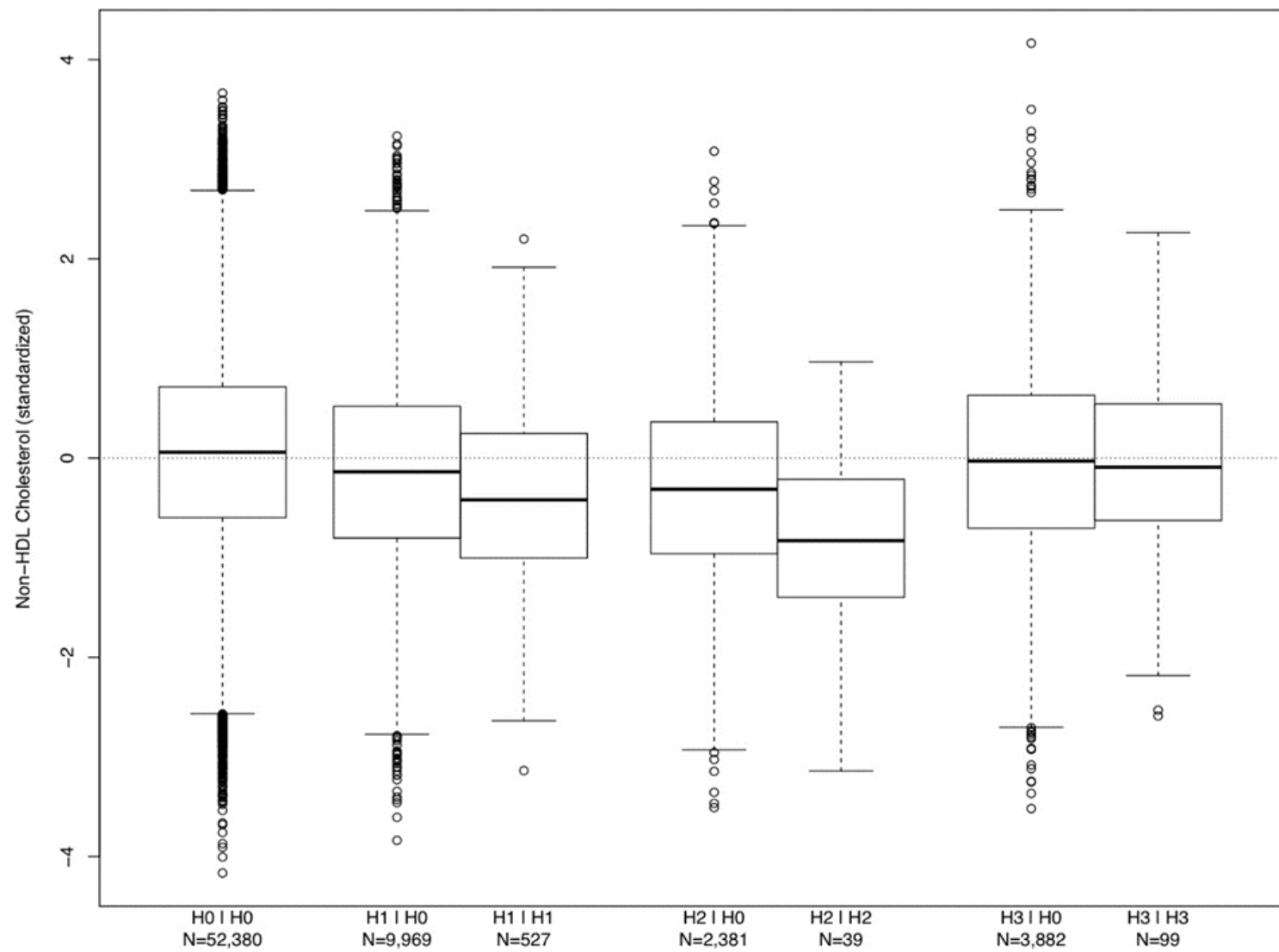

Supplement: S2 Fig — No combination of the minor alleles of these three variants occur on the same haplotype. We denote the four possible haplotypes by H0 (major allele for all three variants), H1 (carrying minor allele of upstream variant rs17248720-T), H2 (carrying minor allele of splice region variant rs72658867-A) and H3 (carrying minor allele of intronic variant rs17248748-T). (PDF) [file pgen.1005379.s002.pdf]

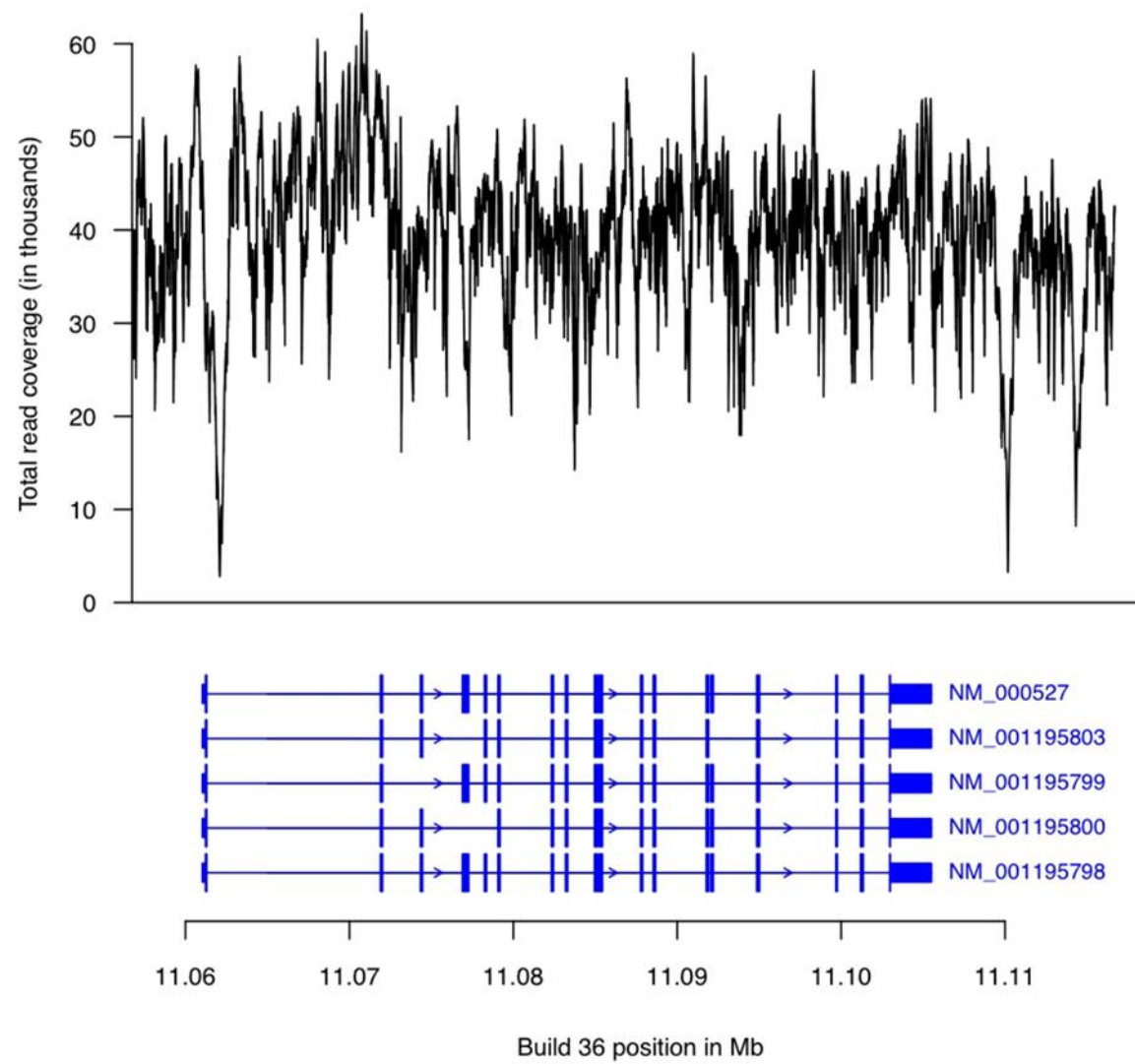

Supplement: S3 Fig — The structure of all LDLR RefSeq transcripts variants are shown in panel below. (PDF) [file pgen.1005379.s003.pdf]

a) Blood

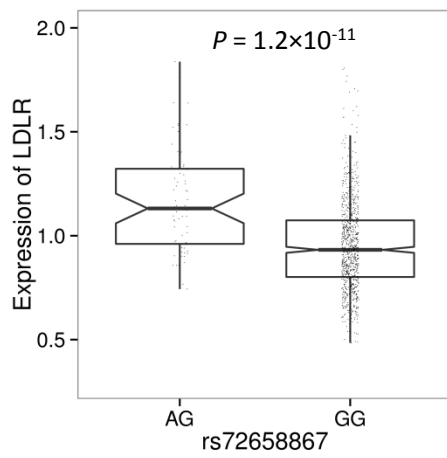

b) Adipose

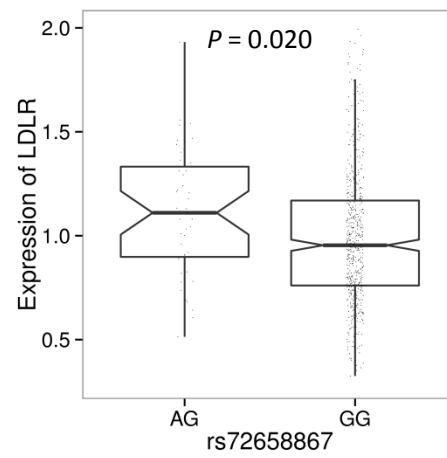

Supplement: S4 Fig — Expression of LDLR in (a) blood and (b) adipose tissue in rs72658867 non-carriers (GG) and carriers (AG). P-values are from the regression of the average log expression ratio on the carrier status, adjusting for age and sex, and differential counts for blood. (PDF) [file pgen.1005379.s004.pdf]

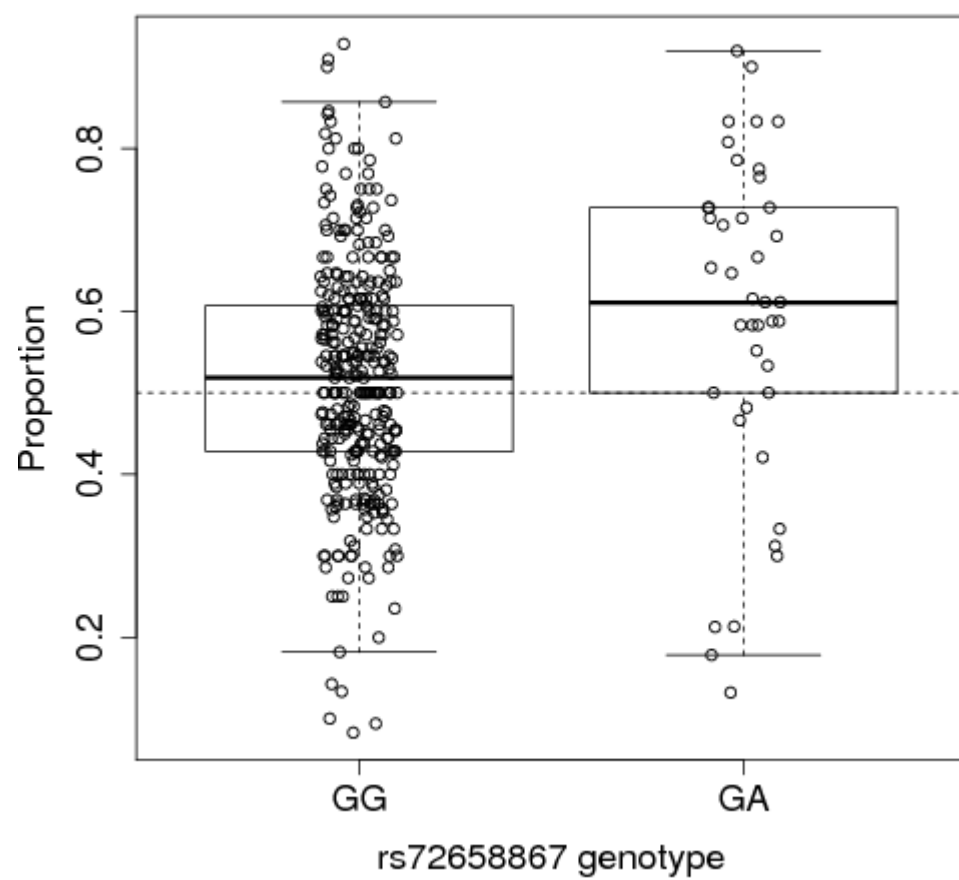

Supplement: S5 Fig — Shown is the proportion of read bases for the synonymous variants in heterozygous state for rs72658867 GG and GA carriers, where the numerator is base count for the allele correlated with rs72658867[A]. Median proportion for rs72658867 GG and GA carriers are 0.52 and 0.61, respectively (P = 0.0016, Mann-Whitney test). Broken line corresponds to proportion of 0.50; the median proportion for the non-carriers being higher reflects reference mapping bias of RNA-Seq reads. (PDF) [file pgen.1005379.s005.pdf]

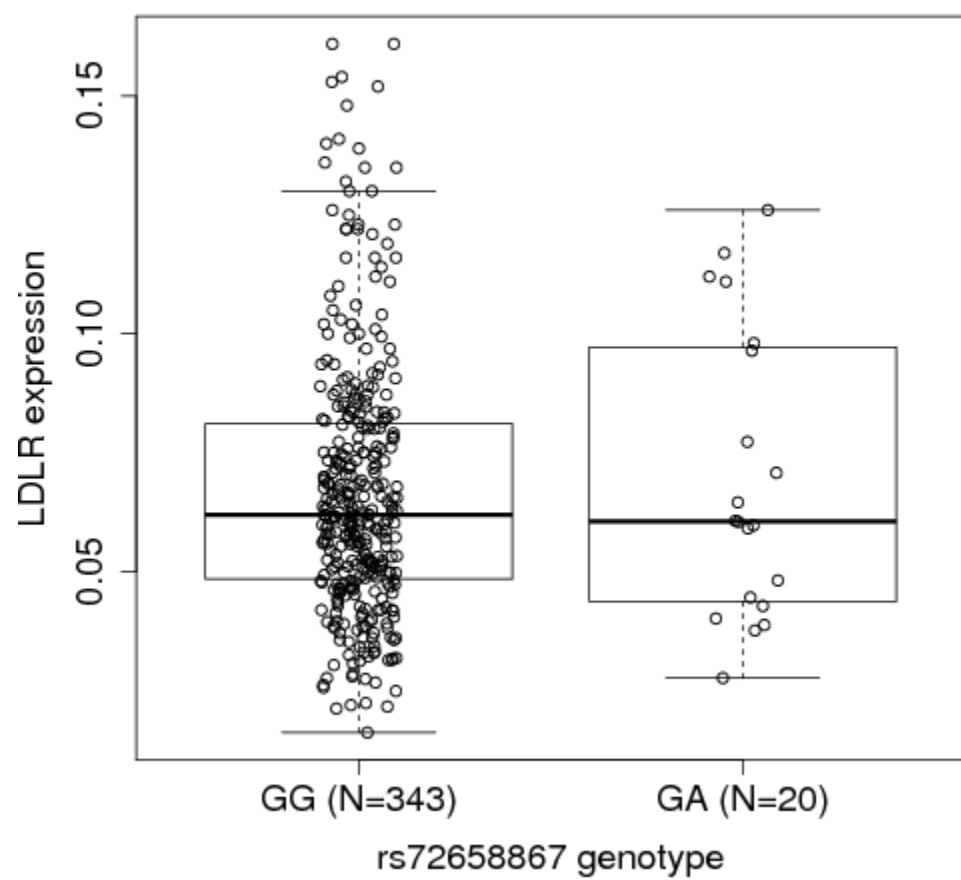

Supplement: S6 Fig — Boxplots show LDLR expression relative to beta-actin for rs72658867 in non-carriers (median: 0.062) and heterozygotes (median: 0.061). No significant difference in expression was found for the two groups (Mann-Whitney test: P = 0.87). (PDF) [file pgen.1005379.s006.pdf]
