# Supplementary material for: A Splice Region Variant in LDLR Lowers Non-high Density Lipoprotein Cholesterol and Protects against Coronary Artery Disease
Source: PLoS Genet. 2015 Sep 1;11(9):e1005379. doi: 10.1371/journal.pgen.1005379 (PMC4556698; doi:10.1371/journal.pgen.1005379)
Supplement: S2 Table — Association results for rs17248720, rs17248748, rs200238879 and rs72658867 with non-HDL-C and LDL-C. Association results for each variant is presented with and without adjusting for the other three variants in the table. a Freq A1 = allellic frequency for allele A1. b Info = imputation quality score. c Effect (β) in mmol/l is given with respect to the allele A1. (DOCX) [file pgen.1005379.s009.docx]

| **S2 Table. Association of *LDLR* sequence variants with non-HDL-C and LDL-C in Iceland** | | | | | | | | | | | | | | | | | | | | |
| --- | --- | --- | --- | --- | --- | --- | --- | --- | --- | --- | --- | --- | --- | --- | --- | --- | --- | --- | --- | --- |
|  | |  |  |  |  |  |  |  | **non-HDL-C (mmol/l) (n=119,146)** | | | |  | **LDL-C (mmol/l) (n=53,841)** | | | | |  |  |
|  | |  |  |  |  |  |  |  | **unadjusted** | | **adjusted** | |  | **unadjusted** | | | **adjusted** | |  |  |
| **Marker** | | **chr19 pos (hg18)** | **A1** | **A2** | **Freq A1** ^a^ **[%]** | **Info** ^b^ | ***LDLR* context** |  | ***P*** | **β**^c^ | ***P*** | **β**^c^ |  | ***P*** | **β**^c^ | | ***P*** | **β**^c^ |  |  |
| rs17248720 | | 11,059,187 | T | C | 8.8 | 0.99 | upstream |  | 2.6E-72 | -0.23 | 1.8E-80 | -0.24 |  | 2.5E-66 | | -0.23 | 6.7E-74 | -0.25 |  |  |
| rs17248748 | | 11,067,040 | T | C | 3.4 | 0.99 | intronic |  | 5.7E-07 | -0.10 | 5.0E-11 | -0.13 |  | 4.0E-06 | | -0.10 | 6.3E-10 | -0.13 |  |  |
| rs200238879 | | 11,077,278 | C | T | 0.06 | 0.95 | splice donor |  | 1.2E-23 | 1.39 | 2.2E-22 | 1.33 |  | 5.0E-20 | | 1.28 | 7.3E-19 | 1.23 |  |  |
| rs72658867 | | 11,092,203 | A | G | 2.2 | 0.98 | splice region |  | 5.2E-63 | -0.42 | 2.0E-70 | -0.44 |  | 1.8E-53 | | -0.41 | 2E-60 | -0.43 |  |  |
|  |  |  |  |  |  |  |  |  |  |  |  |  |  |  |  |  |  |  |  |  |
| Association results for rs17248720, rs17248748, rs200238879 and rs72658867 with non-HDL-C and LDL-C. Association results for each variant is presented with and without adjusting for the other three variants in the table. ^a^ Freq A1=allelic frequency for allele A1. ^b^ Info=imputation quality information.  ^c^ Effect (β) in mmol/l is given with respect to the allele A1. | | | | | | | | | | | | | | | | | | | | |
