# Supplementary material for: A Splice Region Variant in LDLR Lowers Non-high Density Lipoprotein Cholesterol and Protects against Coronary Artery Disease
Source: PLoS Genet. 2015 Sep 1;11(9):e1005379. doi: 10.1371/journal.pgen.1005379 (PMC4556698; doi:10.1371/journal.pgen.1005379)
Supplement: S3 Table — f1 and f2 are the allele frequencies for variants 1 and 2, respectively. β1 and β2 correspond to (adjusted) effects on non-HDL-C with respect to variants 1 and 2. Sign of correlation is with respect to the alleles of the variants. Calculations are based on 104,202 imputed Icelandic individuals that have phased genotypes. (DOCX) [file pgen.1005379.s010.docx]

**S3 Table: D' and r^2^ between the four non-HDL-C associated variants at LDLR.**

|  |  |  |  | non-HDL-C (mmol/l) | |  |  | sign of correlation |  |
| --- | --- | --- | --- | --- | --- | --- | --- | --- | --- |
| Variant 1 | Variant 2 | f_1_ [%] | f_2_ [%] | β_1_ | β_2_ |  | r^2^ |  | D' |
| rs17248720-T | rs72658867-A | 8.8 | 2.2 | -0.24 | -0.44 |  | 0.0022 | -1 | 1.00 |
| rs17248720-T | rs200238879-C | 8.8 | 0.06 | -0.24 | 1.33 |  | 0.000058 | -1 | 1.00 |
| rs17248720-T | rs17248748-T | 8.8 | 3.4 | -0.24 | -0.13 |  | 0.0034 | -1 | 1.00 |
| rs72658867-A | rs200238879-C | 2.2 | 0.06 | -0.44 | 1.33 |  | 0.000013 | -1 | 1.00 |
| rs72658867-A | rs17248748-T | 2.2 | 3.4 | -0.44 | -0.13 |  | 0.00080 | -1 | 1.00 |
| rs200238879-C | rs17248748-T | 0.06 | 3.4 | 1.33 | -0.13 |  | 0.000021 | -1 | 1.00 |

f_1_ and f_2_ are the allele frequencies for variants 1 and 2, respectively.

β_1_ and β_2_ correspond to (adjusted) effects on non-HDL-C with respect to variants 1 and 2.

Sign of correlation is with respect to the alleles of the variants.

Calculations are based on 104,202 imputed Icelandic individuals that have phased genotypes.
