# Supplementary material for: A Splice Region Variant in LDLR Lowers Non-high Density Lipoprotein Cholesterol and Protects against Coronary Artery Disease
Source: PLoS Genet. 2015 Sep 1;11(9):e1005379. doi: 10.1371/journal.pgen.1005379 (PMC4556698; doi:10.1371/journal.pgen.1005379)
Supplement: S4 Table — *We denote the four possible haplotypes by H0 (major allele for all three variants), H1 (carrying minor allele of upstream variant rs17248720-T), H2 (carrying minor allele of splice region variant rs72658867-A) and H3 (carrying minor allele of intronic variant rs17248748-T). The notation Hn|Hm stands for the individual that carries haplotype Hn on one chromosome and Hm on the other (n,m = 0,1,2,3). (DOCX) [file pgen.1005379.s011.docx]

**S4 Table: Non-HDL levels for individuals with phased and imputed genotypes for the three non-HDL lowering variants rs17248720-T, rs72658867-A and rs17248748-T**

|  |  | **Non-HDL levels (standardized units)** | |
| --- | --- | --- | --- |
| **Genotype*** | ***N*** | **Mean** | **95%CI** |
| H0\|H0 | 51,692 | 0.062 | (0.054, 0.070) |
|  |  |  |  |
| H1\|H0 | 9,969 | -0.139 | (-0.158, -0.120) |
| H1\|H1 | 527 | -0.388 | (-0.467, -0.309) |
|  |  |  |  |
| H2\|H0 | 2,381 | -0.311 | (-0.351, -0.271) |
| H2\|H2 | 39 | -0.826 | (-1.107, -0.545) |
|  |  |  |  |
| H3\|H0 | 3,882 | -0.050 | (-0.081, -0.019) |
| H3\|H3 | 99 | -0.117 | (-0.301, 0.068) |
|  |  |  |  |
| H1\|H2 | 253 | -0.492 | (-0.612, -0.373) |
| H1\|H3 | 354 | -0.280 | (-0.383, -0.177) |
| H2\|H3 | 81 | -0.509 | (-0.708, -0.311) |

*We denote the four possible haplotypes by H0 (major allele for all three variants), H1 (carrying minor allele of upstream variant rs17248720-T), H2 (carrying minor allele of splice region variant rs72658867-A) and H3 (carrying minor allele of intronic variant rs17248748-T). The notation Hn|Hm stands for the individual that carries haplotype Hn on one chromosome and Hm on the other.
