# Supplementary material for: A Splice Region Variant in LDLR Lowers Non-high Density Lipoprotein Cholesterol and Protects against Coronary Artery Disease
Source: PLoS Genet. 2015 Sep 1;11(9):e1005379. doi: 10.1371/journal.pgen.1005379 (PMC4556698; doi:10.1371/journal.pgen.1005379)
Supplement: S6 Table — Association results for rs17248720, rs17248748, rs200238879 and rs72658867 with lifespan. Lifespan corresponds to individuals born after 1890 that lived to be 50 years old. Effect (β) in years is given with respect to the allele A1. Each variant is adjusted for the other three variants in the table. (DOCX) [file pgen.1005379.s013.docx]

**S6 Table: Association of *LDLR* sequence variants with lifespan**

|  |  |  |  |  |  |  | **Lifespan** | | | | |
| --- | --- | --- | --- | --- | --- | --- | --- | --- | --- | --- | --- |
|  |  |  |  |  |  |  | **unadjusted** | |  | **adjusted** | |
| **Marker** | **chr19 pos (hg18)** | **A1** | **A2** | **Freq A1 [%]** | **LDLR context** |  | ***P*** | **β** |  | ***P*** | **β** |
| rs17248720 | 11,059,187 | T | C | 8.8 | upstream |  | 4.2E-04 | 0.59 |  | 2.8E-04 | 0.61 |
| rs17248748 | 11,067,040 | T | C | 3.4 | intronic |  | 0.75 | 0.08 |  | 0.55 | 0.16 |
| rs200238879 | 11,077,278 | C | T | 0.06 | splice donor |  | 0.0010 | -6.48 |  | 0.0010 | -6.46 |
| rs72658867 | 11,092,203 | A | G | 2.2 | splice region |  | 0.091 | 0.54 |  | 0.064 | 0.59 |

Association results for rs17248720, rs17248748, rs200238879 and rs72658867 with lifespan. Lifespan corresponds to individuals born after 1890 that lived to be 50 years old. Effect (β) in years is given with respect to the allele A1. Each variant is adjusted for the other three variants in the table.
