# Supplementary material for: A Splice Region Variant in LDLR Lowers Non-high Density Lipoprotein Cholesterol and Protects against Coronary Artery Disease
Source: PLoS Genet. 2015 Sep 1;11(9):e1005379. doi: 10.1371/journal.pgen.1005379 (PMC4556698; doi:10.1371/journal.pgen.1005379)
Supplement: S7 Table — aEffect (β) is given with respect to the allele A1. bThe index variant rs17248720 is included in the table. (DOCX) [file pgen.1005379.s014.docx]

**S7 Table: Variants that have r^2^>0.8 with the upstream variant rs17248720-T in a 2Mb window around it**

|  | **chr19 pos.** |  |  | **Freq.** |  |  | **Gene** | **r^2^ with** | **Association with non-HDL** | |
| --- | --- | --- | --- | --- | --- | --- | --- | --- | --- | --- |
| **Marker** | **[hg18]** | **A1** | **A2** | **A1 [%]** | **Info** | **Gene** | **context** | **rs17248720** | ***P*** | **β^a^ [SD]** |
| rs143020224 | 11,048,324 | G | C | 8.84 | 0.99 | NA | NA | 0.995 | 2.6E-71 | -0.19 |
| rs144826254 | 11,048,358 | G | T | 8.85 | 1.00 | NA | NA | 0.996 | 6.0E-71 | -0.19 |
| rs112736558 | 11,048,422 | C | T | 8.65 | 0.99 | NA | NA | 0.971 | 1.9E-71 | -0.20 |
| rs111989435 | 11,048,611 | G | A | 8.87 | 1.00 | NA | NA | 0.996 | 4.1E-71 | -0.19 |
| rs55997232 | 11,049,117 | T | C | 8.86 | 1.00 | NA | NA | 0.996 | 6.1E-71 | -0.19 |
| rs55791371 | 11,049,153 | C | A | 8.86 | 1.00 | NA | NA | 0.996 | 5.9E-71 | -0.19 |
| rs56125973 | 11,049,164 | C | T | 8.87 | 1.00 | NA | NA | 0.996 | 3.2E-71 | -0.19 |
| rs56289821 | 11,049,247 | A | G | 8.86 | 1.00 | NA | NA | 0.996 | 7.8E-71 | -0.19 |
| rs145329186 | 11,049,313 | CTTTA | C | 8.88 | 0.99 | NA | NA | 0.996 | 2.3E-71 | -0.19 |
| chr19:11049544:0:C | 11,049,544 | C | !C | 9.73 | 0.96 | NA | NA | 0.932 | 1.1E-68 | -0.19 |
| rs112898275 | 11,049,850 | C | T | 8.82 | 0.99 | NA | NA | 0.992 | 9.3E-71 | -0.19 |
| rs112374545 | 11,049,899 | T | C | 8.56 | 0.95 | NA | NA | 0.872 | 7.4E-59 | -0.18 |
| chr19:11049974:I | 11,049,974 | A | AC | 8.55 | 0.98 | NA | NA | 0.940 | 5.5E-70 | -0.20 |
| rs201579954 | 11,050,047 | T | TA | 8.78 | 0.99 | NA | NA | 0.986 | 1.6E-71 | -0.20 |
| rs148898583 | 11,050,205 | G | C | 8.83 | 0.99 | NA | NA | 0.994 | 1.1E-70 | -0.19 |
| rs113722226 | 11,050,272 | C | T | 8.83 | 0.99 | NA | NA | 0.993 | 1.1E-71 | -0.19 |
| rs200495339 | 11,050,298 | C | CG | 8.83 | 0.99 | NA | NA | 0.994 | 2.2E-71 | -0.19 |
| rs12984881 | 11,050,362 | TA | T | 8.84 | 0.99 | NA | NA | 0.995 | 4.9E-71 | -0.19 |
| rs201408139 | 11,050,366 | CAG | C | 8.83 | 0.99 | NA | NA | 0.992 | 2.7E-70 | -0.19 |
| rs73015011 | 11,050,764 | C | T | 8.87 | 1.00 | NA | NA | 0.996 | 3.3E-71 | -0.19 |
| rs114821903 | 11,050,937 | A | T | 8.86 | 1.00 | NA | NA | 0.996 | 3.1E-71 | -0.19 |
| rs138175288 | 11,050,980 | A | C | 8.86 | 1.00 | NA | NA | 0.995 | 2.8E-71 | -0.19 |
| rs112107114 | 11,051,074 | A | G | 8.87 | 1.00 | NA | NA | 0.996 | 2.8E-71 | -0.19 |
| rs115594766 | 11,051,110 | G | A | 8.84 | 1.00 | NA | NA | 0.993 | 6.1E-71 | -0.19 |
| rs112032422 | 11,051,292 | C | T | 8.86 | 1.00 | NA | NA | 0.996 | 6.5E-71 | -0.19 |
| chr19:11051457:0:G | 11,051,457 | G | !G | 8.75 | 0.98 | NA | NA | 0.972 | 1.8E-68 | -0.19 |
| rs77265569 | 11,051,481 | T | G | 8.89 | 0.99 | NA | NA | 0.994 | 8.9E-71 | -0.19 |
| rs142158911 | 11,051,534 | A | G | 8.85 | 0.99 | NA | NA | 0.991 | 6.4E-71 | -0.19 |
| rs118068660 | 11,051,544 | T | C | 8.84 | 0.99 | NA | NA | 0.988 | 8.0E-71 | -0.19 |
| rs145960625 | 11,051,549 | A | G | 8.83 | 0.99 | NA | NA | 0.992 | 3.0E-71 | -0.19 |
| rs139853365 | 11,051,556 | C | T | 8.86 | 1.00 | NA | NA | 0.996 | 2.5E-71 | -0.19 |
| rs142130958 | 11,051,652 | A | G | 8.86 | 1.00 | NA | NA | 0.996 | 6.4E-71 | -0.19 |
| rs73015013 | 11,051,873 | T | C | 8.87 | 1.00 | NA | NA | 0.996 | 4.1E-71 | -0.19 |
| rs114846969 | 11,052,197 | A | G | 8.86 | 1.00 | NA | NA | 0.995 | 1.5E-70 | -0.19 |
| chr19:11052201:I | 11,052,201 | AG | A | 8.86 | 1.00 | NA | NA | 0.996 | 1.4E-70 | -0.19 |
| rs73015016 | 11,052,300 | A | G | 8.87 | 1.00 | NA | NA | 0.996 | 4.1E-71 | -0.19 |
| rs10402112 | 11,052,677 | A | T | 8.87 | 1.00 | NA | NA | 0.996 | 3.9E-71 | -0.19 |
| rs138294113 | 11,052,729 | T | C | 8.86 | 1.00 | NA | NA | 0.995 | 8.2E-71 | -0.19 |
| rs61194703 | 11,053,193 | T | A | 8.87 | 1.00 | NA | NA | 0.996 | 4.3E-71 | -0.19 |
| rs73015020 | 11,053,550 | A | G | 8.87 | 1.00 | NA | NA | 0.996 | 4.5E-71 | -0.19 |
| rs77140532 | 11,053,831 | G | A | 8.88 | 1.00 | NA | NA | 0.996 | 2.4E-71 | -0.19 |
| chr19:11053876:0:CT | 11,053,876 | !CT | CT | 9.16 | 0.98 | NA | NA | 0.968 | 3.2E-69 | -0.19 |
| chr19:11053876:0:CTT | 11,053,876 | CTT | !CTT | 8.87 | 0.99 | NA | NA | 0.991 | 3.2E-69 | -0.19 |
| rs73015021 | 11,053,915 | G | A | 8.88 | 1.00 | NA | NA | 0.996 | 2.1E-71 | -0.19 |
| rs112552009 | 11,054,091 | G | T | 8.86 | 1.00 | NA | NA | 0.996 | 2.7E-71 | -0.19 |
| rs10412048 | 11,054,949 | G | A | 8.87 | 1.00 | NA | NA | 0.996 | 2.4E-71 | -0.19 |
| rs139306531 | 11,057,356 | A | AC | 8.85 | 0.99 | *LDLR* | upstream | 0.997 | 9.7E-72 | -0.19 |
| chr19:11057651:0:ATTTTTTT | 11,057,651 | ATTTTTTT | !ATTTTTTT | 10.47 | 0.91 | *LDLR* | upstream | 0.839 | 4.8E-64 | -0.18 |
| rs8106503 | 11,057,886 | C | T | 8.84 | 0.99 | *LDLR* | upstream | 1.000 | 4.0E-72 | -0.20 |
| rs12151108 | 11,058,261 | A | G | 8.85 | 0.99 | *LDLR* | upstream | 0.997 | 9.8E-72 | -0.19 |
| rs73015024 | 11,058,598 | T | G | 8.75 | 0.99 | *LDLR* | upstream | 0.987 | 5.4E-72 | -0.20 |
| rs75124224 | 11,058,750 | GA | G | 8.87 | 0.99 | *LDLR* | upstream | 0.997 | 1.1E-71 | -0.19 |
| rs17248720^b^ | 11,059,187 | T | C | 8.84 | 0.99 | *LDLR* | upstream | 1.000 | 2.6E-72 | -0.20 |
| rs17248727 | 11,059,502 | C | T | 8.85 | 0.99 | *LDLR* | upstream | 0.997 | 8.7E-72 | -0.19 |
| rs60173709 | 11,062,988 | G | GT | 8.88 | 0.99 | *LDLR* | intronic | 0.996 | 3.6E-72 | -0.20 |
| rs141787760 | 11,063,194 | G | GC | 8.88 | 0.99 | *LDLR* | intronic | 0.996 | 3.3E-72 | -0.20 |
| rs6511720 | 11,063,306 | T | G | 8.88 | 0.99 | *LDLR* | intronic | 0.996 | 3.7E-72 | -0.20 |

| ^a^Effect (β) is given with respect to the allele A1. |
| --- |
| ^b^The index variant rs17248720 is included in the table. |
