# Supplementary material for: A Splice Region Variant in LDLR Lowers Non-high Density Lipoprotein Cholesterol and Protects against Coronary Artery Disease
Source: PLoS Genet. 2015 Sep 1;11(9):e1005379. doi: 10.1371/journal.pgen.1005379 (PMC4556698; doi:10.1371/journal.pgen.1005379)
Supplement: S8 Table — aβ given with respect to A1, bthe two top eQTL variants rs72658867 and rs180760728 are correlated (r2 = 0.89). The non-HDL-C association for the second best eQTL variant rs180760728 is not significant after adjusting for rs72658867 (P adj = 0.32, βadj = 0.32). The non-HDL-C association for rs72658867 remains significant once adjusted for the second best eQTL variant (P adj = 8.7e-11, βadj = -0.41). (DOCX) [file pgen.1005379.s015.docx]

**S8 Table: Markers with eQTL *P*<1e-4 in white blood cells for LDLR probe (NM_000527) in the *LDLR* region.**

| **Marker** | **chr19 pos. [hg18]** | **A1** | **A2** | **Freq. A1 [%]** | **Info** | **Gene** | **gene context** | ***P*** | **β^a^** |
| --- | --- | --- | --- | --- | --- | --- | --- | --- | --- |
| chr19:10893164:S | 10,893,164 | A | G | 0.43 | 0.99 | *CARM1* | intronic | 4.6E-05 | 1.18 |
| rs73013141 | 10,959,309 | G | A | 1.17 | 0.98 | *SMARCA4* | intronic | 7.8E-05 | 0.75 |
| rs73013176 | 11,008,526 | C | T | 2.37 | 1.00 | *SMARCA4* | intronic | 1.1E-05 | 0.58 |
| rs180760728^b^ | 11,080,281 | C | T | 1.98 | 0.98 | *LDLR* | intronic | 6.5E-08 | 0.71 |
| rs72658867^b^ | 11,092,203 | A | G | 2.22 | 0.98 | *LDLR* | splice region | 2.4E-08 | 0.72 |
| chr19:11094071:0:CT | 11,094,071 | !CT | CT | 29.61 | 0.96 | *LDLR* | intronic | 2.6E-05 | 0.21 |
| rs11557092 | 11,118,018 | T | C | 24.93 | 0.99 | *SPC24* | downstream | 3.2E-05 | 0.22 |
| rs79050709 | 11,135,530 | T | C | 11.05 | 0.99 | *KANK2* | downstream | 3.7E-05 | 0.28 |
| chr19:11184999:S | 11,184,999 | T | C | 1.34 | 0.97 | *DOCK6* | synonymous | 5.2E-05 | 0.70 |
| rs183236769 | 11,240,282 | A | G | 1.36 | 0.97 | - | - | 4.5E-05 | 0.72 |

| ^a^β given with respect to A1 |
| --- |
| ^b^The two top eQTL variants rs72658867 and rs180760728 are correlated (r^2^=0.89). |
| The non-HDL-C association for the second best eQTL variant rs180760728 is not significant after adjusting for rs72658867 (P_adj_=0.32, β_adj_=0.32). |
| The non-HDL-C association for rs72658867 remains significant once adjusted for the second best eQTL variant (P_adj_=8.7E-11, β_adj_=-0.41). |
