# Supplementary material for: A Splice Region Variant in LDLR Lowers Non-high Density Lipoprotein Cholesterol and Protects against Coronary Artery Disease
Source: PLoS Genet. 2015 Sep 1;11(9):e1005379. doi: 10.1371/journal.pgen.1005379 (PMC4556698; doi:10.1371/journal.pgen.1005379)
Supplement: S9 Table — Allele specific expression was evaluated with five different allele specific markers. r2, D’ and sign correspond to LD calculations for rs72658867-A and alternative allele (ALT) for the allele specific markers; if sign = 1, then the allele rs72658867-A is correlated with the ALT allele of the marker; if sign = -1, then the allele rs72658867-A is correlated with the reference allele (REF) of the marker. Mean, std. dev and median correspond to the proportion of read bases for the allele specific marker (if sign = 1, proportion = #ALT bases/#total bases; if sign = -1, proportion = #REF bases/#total bases). Δmedian: median rs72658867 GA–median rs72658867 GG. (Note: The median for the rs72658867 heterozygotes is always higher than the median for the non-carriers.) P corresponds to the significance level for testing difference in the read base proportions for the two groups (rs72658867 GA and rs72658867 GG) for the allele specific markers, using a two-sided Mann-Whitney test. (DOCX) [file pgen.1005379.s016.docx]

**S9 Table: Allele specific expression analysis for rs72658867-A heterozygous carriers and non-carriers using base counts at synonymous variants in heterozygous state.**

|  |  |  |  |  | **LD calculations for rs72658867[A] and ALT** | | | **rs72658867 genotype GG** | | | |  | **rs72658867 genotype GA** | | | |  |  |  |
| --- | --- | --- | --- | --- | --- | --- | --- | --- | --- | --- | --- | --- | --- | --- | --- | --- | --- | --- | --- |
| **Allele specific markers** | **chr19 pos. [hg18]** | **REF** | **ALT** | **Freq. Alt[%]** | **r^2^** | **D'** | **sign** | ***N*** | **mean** | **std.dev** | **median** |  | ***N*** | **mean** | **std.dev** | **median** |  | **Δmedian** | ***P*** |
| rs5930 | 11,085,265 | A | G | 57.7 | 0.03 | 1.00 | -1 | 50 | 0.50 | 0.15 | 0.49 |  | 6 | 0.45 | 0.20 | 0.50 |  | 0.02 | 0.56 |
| rs1799898 | 11,088,554 | C | T | 11.5 | 0.18 | 1.00 | 1 | 26 | 0.50 | 0.15 | 0.49 |  | 10 | 0.58 | 0.19 | 0.59 |  | 0.09 | 0.15 |
| rs688 | 11,088,602 | C | T | 45.2 | 0.02 | 1.00 | -1 | 91 | 0.52 | 0.16 | 0.52 |  | 8 | 0.71 | 0.23 | 0.77 |  | 0.24 | 0.0058 |
| rs5925 | 11,091,881 | T | C | 45.1 | 0.02 | 1.00 | -1 | 88 | 0.52 | 0.14 | 0.54 |  | 6 | 0.58 | 0.21 | 0.63 |  | 0.09 | 0.10 |
| rs5927 | 11,094,941 | A | G | 77.0 | 0.08 | 0.99 | -1 | 57 | 0.53 | 0.14 | 0.50 |  | 11 | 0.61 | 0.17 | 0.62 |  | 0.12 | 0.12 |

Allele specific expression was evaluated with five different allele specific markers. r^2^, D’ and sign correspond to LD calculations for rs72658867-A and alternative allele (ALT) for the allele specific markers; if sign=1, then the allele rs72658867-A is correlated with the ALT allele of the marker; if sign=-1, then the allele rs72658867-A is correlated with the reference allele (REF) of the marker.

Mean, std. dev and median correspond to the proportion of read bases for the allele specific marker (if sign=1, proportion=#ALT bases/#total bases; if sign=-1, proportion=#REF bases/#total bases).

Δmedian: median rs72658867 GA – median rs72658867 GG. (Note: The median for the rs72658867 heterozygotes is always higher than the median for the non-carriers.)

*P* corresponds to the significance level for testing difference in the read base proportions for the two groups (rs72658867 GA and rs72658867 GG) for the allele specific markers, using a two-sided Mann-Whitney test.
