# Supplementary material for: A Splice Region Variant in LDLR Lowers Non-high Density Lipoprotein Cholesterol and Protects against Coronary Artery Disease
Source: PLoS Genet. 2015 Sep 1;11(9):e1005379. doi: 10.1371/journal.pgen.1005379 (PMC4556698; doi:10.1371/journal.pgen.1005379)
Supplement: S10 Table — The mean ratio of coverages for intron 14 vs. exon 14 is 0.21, indicating an intron retention. To quantitate the proportion of transcripts with intron 14 retention we consider the proportion of RNA sequencing reads that are spliced from exon 14 to exon 15 (correctly spliced) out of the total number of reads that cover the last base of exon 14; the mean proportion of reads spliced is 0.49, indicating that about half of the LDLR transcripts are incorrectly spliced. (DOCX) [file pgen.1005379.s017.docx]

**S10 Table: Analysis of splicing of exons 14 and 15 in *LDLR* based on RNA-seq from three blood donors who are homozygous for the splice region variant rs72658867-A**

|  | **Average read coverage** | | |  | **Spliced reads for exon 14 to exon 15** | | |
| --- | --- | --- | --- | --- | --- | --- | --- |
| **Sample** | **Exon 14** | **Intron 14** | **Ratio of coverages (intron 14 vs exon 14)** |  | **#spliced reads** | **#reads covering last base of exon 14 reading into intron 14** | **Proportion reads spliced** |
| 1 | 305 | 65 | 0.21 |  | 155 | 143 | 0.52 |
| 2 | 455 | 106 | 0.23 |  | 172 | 296 | 0.37 |
| 3 | 219 | 39 | 0.18 |  | 118 | 81 | 0.59 |

The mean ratio of coverages for intron 14 vs. exon 14 is 0.21, indicating an intron retention. To quantitate the proportion of transcripts with intron 14 retention we consider the proportion of RNA sequencing reads that are spliced from exon 14 to exon 15 (correctly spliced) out of the total number of reads that cover the last base of exon 14; the mean proportion of reads spliced is 0.49, indicating that about half of the LDLR transcripts are incorrectly spliced.
