# Supplementary material for: A Splice Region Variant in LDLR Lowers Non-high Density Lipoprotein Cholesterol and Protects against Coronary Artery Disease
Source: PLoS Genet. 2015 Sep 1;11(9):e1005379. doi: 10.1371/journal.pgen.1005379 (PMC4556698; doi:10.1371/journal.pgen.1005379)
Supplement: S7 Fig — A schematic diagram of the domain structure of the LDLR protein is shown at the top (domains deleted from the truncated receptor are filled). The wild type nucleotide and amino acid sequence for the end of exon 14 and start of exon 15 is shown in the middle. At the bottom: the nucleotide sequence of the abnormally spliced RNA (in red intron 14 retention, in bold is the splice site mutation, c.2140+5G>A) and the amino acid sequence of the truncated receptor (in blue 33 novel amino acids). (PDF) [file pgen.1005379.s007.pdf]

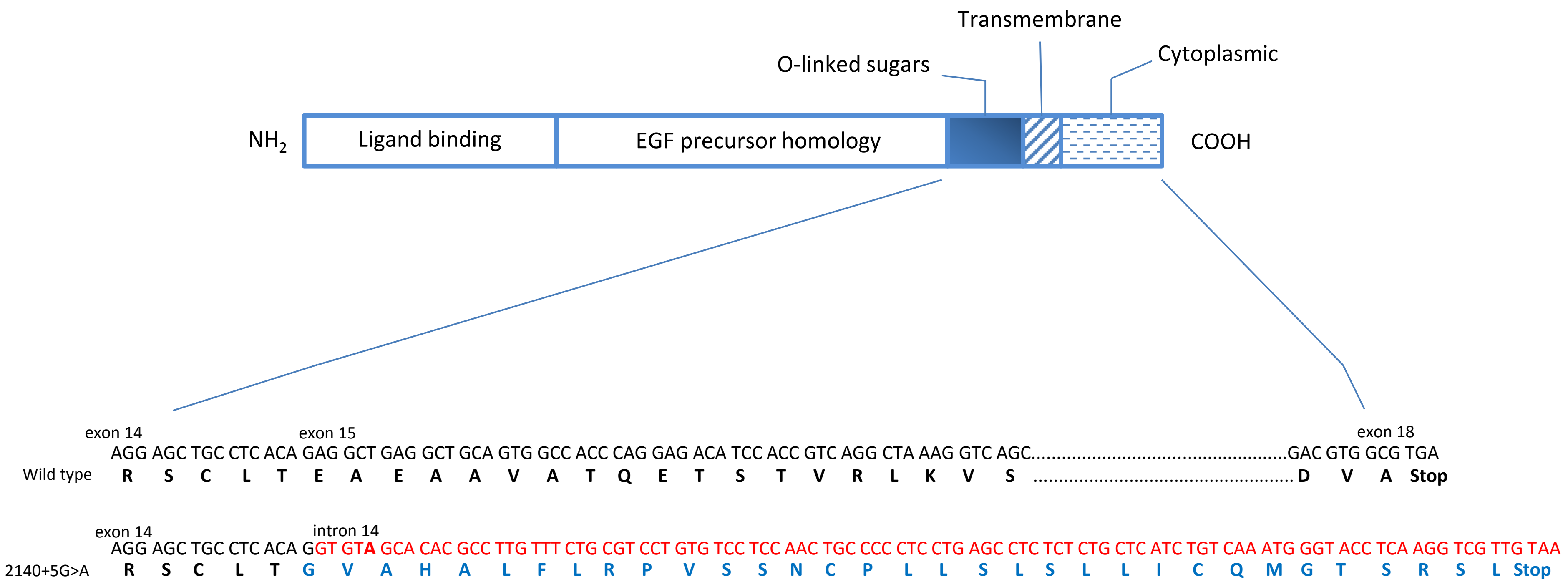
Transmembrane
O-linked sugars
Cytoplasmic
NH2
Ligand binding
EGF precursor homology
COOH
exon 14
exon 15
exon 18
AGG AGC TGC CTC ACA GAG GCT GAG GCT GCA GTG GCC ACC CAG GAG ACA TCC ACC GTC AGG CTA AAG GTC AGC...GAC GTG GCG TGA
Wild type
R S C L T E A E A A V A T Q E T S T V R L K V S .... D V A Stop
exon 14
intron 14
AGG AGC TGC CTC ACA GGT GTA GCA CAC GCC TTG TTT CTG CGT CCT GTG TCC TCC AAC TGC CCC CTC CTG AGC CTC TCT CTG CTC ATC TGT CAA ATG GGT ACC TCA AGG TCG TTG TAA
2140+5G>A
R S C L T G V A H A L F L R P V S S N C P L L S L S L L I C Q M G T S R S L Stop
